# A nationwide study of the impact of social quality factors on life satisfaction among older adults in rural China

**DOI:** 10.1038/s41598-024-61398-4

**Published:** 2024-05-21

**Authors:** Yanfang Xia, Guoyong Wang, Fan Yang

**Affiliations:** 1 School of Sociology and Public Administration, Guizhou Minzu University, Guizhou, China; 2 Law School, Southwest Petroleum University, Sichuan, China; 3 Social Welfare Program, School of Public Administration, Dongbei University of Finance and Economics, 219 Quanxue Hall, 217 Jianshan Street, Shaohekou District, Dalian, 116025 China

**Keywords:** Public health, Quality of life

## Abstract

In rural China, the aging population faces unique challenges that affect their life satisfaction. These challenges are compounded by disparities in access to resources compared to urban areas, making it crucial to address these issues to ensure a dignified and fulfilling later life. Understanding and improving life satisfaction for this demographic is essential, not only for the well-being of the elderly but also for the overall social stability and economic sustainability of these communities. Using a rural sample from the Chinese Social Survey (CSS), this study investigates the interplay between social quality factors and life satisfaction among older adults in rural China. It focuses on the roles of socioeconomic security, social cohesion, social inclusion, and social empowerment in shaping the well-being of this demographic. It was found that the four conditional factors of social quality—socioeconomic security, social cohesion, social inclusion, and social empowerment—have different influences on life satisfaction among older adults in rural areas. The statistical model shows that the influence of absolute family income on social and economic security does not have statistical significance. In contrast, housing, pension insurance participation, and public safety perception positively affect life satisfaction among older adults in rural areas. Among the social cohesion factors, higher social morality, legal system evaluation, social identity, grassroots government, and interpersonal trust contribute to the life satisfaction of older adults in rural areas. Regarding social inclusion factors, good social tolerance, social equity, and perception of government public services can significantly improve life satisfaction among older adults in rural areas. For social empowerment factors, social participation helps expand the social support network of older adults in rural areas, enhancing their life satisfaction. Paths to improving the quality of rural society should be explored to improve relative poverty in rural areas. They should continue to be pursued to strengthen the old-age security system for older adults in rural areas. Further, the National Medium- and Long-Term Plan for Actively Responding to Population Aging should be the base for forming an elderly-friendly society. A good atmosphere promotes the participation of people, families, and society; supports social cohesion; enhances inclusion; and promotes social participation, improving life satisfaction.

Population aging continues to accelerate, and the proportion of China's elderly population is increasing. The increasing cost of old-age care has brought new challenges to welfare policies. The problem of population aging will continue to form a joint challenge with economic and social development issues for a long time. Because most older adults in China live in rural areas, the aging problem is more salient there. Reasonable life satisfaction is an essential manifestation of healthy and active aging. On the one hand, unlike cities, the economic and social development level in China's rural areas is relatively backward, and social security, especially the pension system, is still in its infancy. On the other hand, rural population mobility and social changes have weakened the family's support function. Therefore, it is necessary to examine life satisfaction among older adults in rural areas. How can the life satisfaction of older adults in rural areas be improved to promote the transformation of their resources from single-family support to social resources and to make up for the weakening of traditional family functions? These are urgent problems that must be solved in the process of rural revitalization.

Economic and health status factors are essential factors affecting the life satisfaction of older adults^1^. However, China's rural social security system is still in its infancy, and it remains difficult to improve the life satisfaction of older adults only by enhancing economic security. Economic factors easily bring the climbing effect, accelerating the diminishing marginal impact of happiness^2^. A well-functioning and harmonious society needs economic growth to promote social justice and improve social quality^3^. Economic development that cannot simultaneously improve the social welfare of the entire population is meaningless^3^. As part of a new paradigm of social development research, social quality theory provides a unique perspective for systematically discussing the effect of the external social environment on residents' life satisfaction^4^. Therefore, relative to the fact of "getting old before getting rich" in China, a social quality system should be developed to improve the life satisfaction of older adults as an essential development direction and to increase the value orientation of welfare policies for the elderly in the process of rural revitalization.

The practice of physical activity is one of the variables that should not be overlooked in this area, as it represents an improvement in the quality of life for older adults and a guarantee of successful aging in terms of health^5^. It should be considered as a measure of health protection and functional abilities that result in better quality of life for older adults, leading to better physical health in older adults^6^. It is necessary to consider that among the relevant elements for older people in quality of life, health, social relationships, and staying active are highlighted, with physical activity being one of the most important strategies for disease prevention and health promotion on a physical and cognitive scale^7^.

## Literature review and research hypotheses

Life satisfaction refers to the subjective satisfaction of various needs of societal members^4^. It is an overall evaluation of individual life quality, including housing, economic life, and social connection satisfaction. The subjective satisfaction of the members of a society is a crucial indicator for testing policy effectiveness. Life satisfaction research emerged in the research community in the 1950s, and scholars began to conduct theoretical and empirical research in the 1980s. With the transformation of social development concepts, increasing research shifted to researching the subjective feelings of social members. Scholars in related disciplines have begun to conduct in-depth research on life satisfaction and its influencing factors. A large number of studies have shown that the social environment has a significant influence on life satisfaction^8,9^. Many studies have explored the impacts of the social climate on life satisfaction from the perspectives of social networks, interpersonal relationships, and public policies^10,11^.

The analysis of relevant research results shows that scholars focused on a single factor's influence on life satisfaction in rural China. Previous studies have explored either demographic variables at the individual level, such as gender, age, marital status, number and health of children, personalities, and socioeconomic status, or policy institutions, also at the individual level, of long-term care, social trust, and social security^12^. The research of Yao and colleagues believed that socioeconomic factors had a decisive role in life satisfaction^13^. Another study found that indicators such as household income, owner-occupied housing, and confidence in the future positively impacted life satisfaction^14^. Shen et al. analyzed the impact of socioeconomic factors and four other groups of factors, including work, social equity and mobility, social conflict and public security, and social trust, on the life satisfaction of urban residents^15^. Many valuable studies have been published, but few combined individual and social levels to analyze life satisfaction.

Social quality theory provides a perspective that connects individuals and society. Beck believed that social quality relates to the members of social welfare and their potential and social participation opportunity and ability^16^. A society’s development capacity, openness, and inclusiveness are reflected in its socioeconomic security, opportunities for social participation, and social support obtained by its members^17,18^. Social quality theory focuses on the sociality of individuals, emphasizes the integration and positive interaction between individual development and society^19^, advocates for a close connection between citizens' well-being and social development, and promotes the transformation of social development from GDP doctrine to one that centers on inclusiveness^20^.

Social quality theory’s core structure included constructive, conditional, and normative factors. Researchers usually adopt conditional factors to measure social quality. In contrast, constructive and normative factors are typically used to examine the quality of the system and social development goals. Social quality can be used to measure the living conditions of members of society through the four dimensions of socioeconomic security and social cohesion, inclusion, and empowerment. People should have access to resources that help achieve social interactions, that is, a certain level of socioeconomic security. Another dimension is that social systems and social structures should be open, which means good social inclusion. The third dimension is social cohesion, which refers to collectively recognized values and norms that can promote community formation. Finally, people should be empowered to participate in social interactions, which is the fourth dimension, social empowerment. These four conditional factors refer to material security, collective identity, social justice, and social members’ actions and abilities.

In response to the unique socio-economic and cultural landscape of China, this study has adapted the Social Quality Model to better suit the local conditions. Recognizing the variations in social structure and the specific needs of the rural elderly in China, we have collaborated with scholars from the Chinese Academy of Social Sciences to refine the model's indicators. These modifications include a focused approach on social cohesion, which emphasizes the integration of social values and trust; social inclusion, which now highlights the state’s tolerance towards diverse social behaviors and characteristics, aiming to foster inter-group integration and reduce social conflicts; and social empowerment, redefined to enhance individual mobility within society and encourage active participation in social and political processes. These tailored indicators aim to provide a comprehensive understanding of the social quality in rural China, ensuring that the theoretical framework is not only applicable but also effective in addressing the specific challenges faced in this context. By integrating these adapted indicators, the study contributes to both the theoretical discourse and practical applications of social quality theory, offering a nuanced perspective that is essential for formulating targeted social policies aimed at improving life satisfaction among the rural elderly.

The dimension of socioeconomic security in social quality theory measures the degree to which society provides material and institutional resources to its members^21,22^; in other words, it focuses on the degree to which social members possess or can access resources. Such resources include social members’ means to deal with risks, including economic, living environment, health care, and other aspects. Economic resources, in particular, can improve individuals’ quality of life and material well-being. However, expanding income inequality reduces the happiness experience^23^. A high-quality society should ensure people's living standards and enjoyment of resources such as medical care, old-age security, personal security, social services, and a good environment^24^. The welfare supply that needs to be covered under the indicator system of the socioeconomic security dimension includes the following aspects. First, the basic security of daily life must be included, such as food safety, environmental issues, and a safe work environment. Second, the basic survival security of citizens, such as income, social protection, and health, are necessary to provide. Third, all welfare provisions in liberty, safety, and justice should be recognized^25^.

Social cohesion refers to the cohesion of individuals in social relationships based on shared basic norms, identities, and values^24^. Lin believes that to understand social trust, we should examine interpersonal trust and analyze system (institutional) trust^26^. Social identity is among the indicators reflecting social cohesion, including identity with the country and the region.

Social inclusion measures the extent to which society has removed systemic and nonsystematic barriers to integration^22^, referring to the degree to which people are integrated into the social structure^27^. A society with good social inclusion would not allow its members to suffer unfair treatment due to possessing specific identities or traits^28^.

Social empowerment focuses on social participation ability and opportunities of members of society from the individual level. It pays attention to the extent to which society enhances the potential of social members through the institutional system^26^. For a society with a high degree of empowerment, its social members have sufficient opportunities to participate in positive social interactions. On this basis, their development space can be expanded. The primary measurement content of social empowerment involves social members’ access to knowledge and information, ability, opportunity, willingness, and ability to participate in society^19^.

Previous studies have shown that social quality effectively predicts positive psychology, including happiness and gain^4,29^. However, there is a lack of research systematically investigating the impact of social quality factors on life satisfaction. A limited number of academic literatures has explored the influence of factors such as social support, social participation, and social capital on the life satisfaction of older adults^30^. For example, some studies suggested that social capital contributed to happiness^31,32^, but these factors only related to specific dimensions of social quality and cannot systematically explain the impact of factors of social quality on the life satisfaction.

Among the limited studies that explored the relationship between life satisfaction and certain social quality factors, the focus population is urban residents. There is a severe lack of studies investigating the life satisfaction of older adults in rural areas concerning overall social quality. There are many differences between rural and urban elderly residents. Previous studies revealed the different roles of the four conditional factors of socioeconomic security and social cohesion, inclusion, and empowerment in different regions^33^. Therefore, there is a need to systematically examine the impact of social quality dimensions on the life satisfaction of elderly residents in rural areas.

Considering the gap between urban and rural areas in China's economic and social development, this study comprehensively refers to the European social quality measurement index system and the social quality index system proposed by Chinese scholars^33^. It uses elderly in rural areas samples in the CSS2019 data to quantitatively analyze the impact of social quality on the life satisfaction of rural elderly. Detailed information about the CSS 2019 database could be found in Supplemental file. Focusing on the characteristics of the gap between urban and rural areas in economic and social development, this study comprehensively built a social quality measurement index using the European social quality measurement index system and the social quality index system proposed by Chinese scholars as a reference. We quantitatively analyzed the impact of the four conditional factors of social quality, namely socioeconomic security, social cohesion, social inclusion, and social empowerment, on the life satisfaction of the rural elderly using rural samples from the 2019 CSS data.

From these theoretical observations, this study proposes the following four hypotheses:

### Hypothesis 1

Elderly in rural areas with a higher level of socioeconomic security is more likely to demonstrate a higher level of life satisfaction.

### Hypothesis 2

Elderly in rural areas with a higher level of social cohesion are more likely to demonstrate a higher level of life satisfaction.

### Hypothesis 3

Elderly in rural areas with a higher level of social inclusion are more likely to demonstrate a higher level of life satisfaction.

### Hypothesis 4

Elderly in rural areas with a higher level of social empowerment are more likely to demonstrate higher life satisfaction.

## Research design and variable measurement

### Data sources and samples

The data used in this study are drawn from CSS2019, a large-scale nationwide survey conducted by the Institute of Sociology of the Chinese Academy of Social Sciences. Using the framework of Western social quality theory, CSS2019 focused on contemporary Chinese social quality issues and evaluated the overall development level of Chinese society according to the four main measurement dimensions of social quality (socioeconomic security and social cohesion, inclusion, and empowerment). This dataset has been extensively utilized in a multitude of studies. Researchers have harnessed the rich and diverse data within this dataset to conduct comprehensive tests and analyses, enabling them to gain substantial insights into the various social quality-related phenomena in China^32–34^.

Over 10,000 urban and rural residents were recruited using PPS probability sampling and administrated a household questionnaire. The samples were collected through a four-stage stratified design in which counties/cities/districts were selected. Neighborhood/village committees were then sampled from selected counties/cities/districts, and households were selected from those neighborhood/village committees. Residents were selected at the final stage. Different sampling methods are adopted at each stage. Ultimately, 604 village committees/neighborhood committees in 151 counties/cities/districts were selected, and the data collected were nationally representative. There were 10,283 participants remained after data cleaning.

This study contained the sample data of older adults in rural areas. Only samples with rural household registration and individuals aged ≥ 60 were retained. Finally, 1552 effective samples were analyzed. In the specific analysis process, because of each Variable's different number of effective cases, the number of effective cases used by different models may be less than the sample size. For details, see the number of effective samples listed for each model.

### Variable selection and measurement

#### Dependent variable

The life satisfaction of older adults in rural areas in China should be judged by their perception of their living standards and their overall opinion of rural society as a whole. In this study, the subjective feelings and comprehensive evaluation of older adults in rural areas about their living standards were used as dependent variables, and satisfaction with the overall living conditions in the CSS2019 questionnaire was used to measure this term. Participants gave a value of 1–10 to measure their current life satisfaction.

#### Independent variables

The independent variables of this study are the four conditional factors of social quality: socioeconomic security and social cohesion, inclusion, and empowerment. In selecting sub-indicators of each dimension, this study also refers to the secondary indicators of European scholars’ social quality research and the localization of the Chinese social quality index system, as proposed by Chinese scholars^33^. Because of the complexity of the four conditional factors of social quality, this study uses principal component analysis to reduce the four measurement dimensions of social quality assessed: socioeconomic security and social cohesion, inclusion, and empowerment.

##### Socioeconomic security

According to the actual state of older adults in rural areas, based on the conversion calculation of variables, this study translates socioeconomic security into total household income (logarithmic), housing security, pension insurance participation, and public security perception. Among these factors, housing security is measured using housing conditions. To measure the housing conditions of respondents, the CSS 2019 questionnaire asked, "How many homes do you have at present?" For pension insurance, the questionnaire asks, “Do you have the following social security?” Having a pension is represented with a dummy variable of 1. Public safety perception measures included assessments of the government’s ability to maintain social security and ensure food and drug safety. The answers were provided with five options, from very bad to very good.

##### Social cohesion

This study selects the social trust level, the social moral rule of law level evaluation, and social identity indicators to measure social cohesion. The principal component factor analysis method was used to reduce the dimension of the variables with respect to trust. After the rotation of the maximum variance method, three factors were extracted according to the factor load. These were termed the central government and institutional trust (central government, hospitals, courts, and public security departments), community and market trust (group organizations, charities, news media, banks, and insurance companies), and local government and interpersonal trust (district and county governments, township governments, and people). The KMO value of each item was 0.824, and the Bartlett test showed *p* < 0.001. This is consistent with previous research conclusions, indicating that older adults in rural areas are more inclined to distinguish between the local and central governments^34^.

Social trust factor analysis is shown in Table 1.

**Table 1:** Social trust factor analysis.

Items	Social groups and market trust factor	The central government and institutional trust factor	Local government and general trust factor	Communality
Central government	0.056	0.418	0.191	0.214
District and county governments	0.237	0.125	0.815	0.735
Township government	0.257	0.121	0.826	0.762
Group organizations	0.651	0.066	0.158	0.453
Charities	0.720	− 0.002	0.143	0.539
News media	0.621	0.269	0.058	0.461
Bank	0.592	0.350	− 0.007	0.473
Insurance company	0.603	0.243	0.110	0.434
Hospital	0.259	0.397	0.388	0.375
Court	0.240	0.796	0.099	0.701
Public security department	0.220	0.835	0.121	0.760
Between people	− 0.071	0.135	0.531	0.305
Eigenvalues	3.903	1.285	1.025	6.186
Explanation variance	32.527%	10.710%	8.545%	51.782%

Extraction method: principal component analysis.

Rotation method: Kaiser standardized maximum variance method.

The evaluation of the level of social morality and the rule of law is measured using questionnaire items on the moral status of people in society and the level of people’s compliance with the law in society. The answers included ten options, from very bad to very good, and were assigned 1–5. The higher the score, the higher the evaluation of social morality and the rule of law.

Drawing on the case of China’s rural socioeconomic development, this study used data collected in response to this question, “To what extent do you agree with the following statements?” (1 = strongly disagree, 5 = strongly agree) to measure the degree of the social identity of rural residents. The statements are as follows: (1) I am often proud of my country's achievements; (2) I want to be Chinese again if I have a choice in my next life. (3) Every Chinese has the same opportunity to obtain wealth and happiness; and (4) Without the Chinese Communist Party, Chinawouldfall into chaos. Factor analysis is conducted on these four topics. Bartlett's spherical test is adopted, and maximum variance is used to rotate it. According to the factor load, the typical social identity factor is extracted as the social identity variable. The KMO value between each project is 0.719, and the variance contribution rate is 39.402%.

##### Social inclusion

In this study, the evaluation of social tolerance and equity and the perception of public services are secondary indicators of social inclusion. Concerning the respondents' perception of social tolerance, this study selected the questionnaire: “Please use 1–10 points to express your evaluation of the degree of tolerance in the present society.” The evaluation of social equity measures the response to this question: 'Overall, how do you judge the current situation of social equity in general?' The answers included ten options from very unfair to very fair.

Public service perception was assessed with “Do you think the local government in the following aspects of the work well?” In this question, respondents were asked for their overall perceptions of 11 public services, including health services provided by the central government, social security, environmental protection, protection of citizens’ political rights, punishment of corruption, equity in law enforcement, increasing incomes, increasing employment opportunities, improving transparency in government work, awareness of services, and ensuring equity in education, as well as local government work. The answers included five options, from very bad to very good, with values ranging from 1 to 5. From the factor analysis of these 11 items, the common public service perception factor was extracted according to the factor load; the KMO value was 0.942, the cumulative variance contribution rate was 51.286%, and the Butterritsphericity test was *p* < 0.001.

##### Social empowerment

This study measured older adults’ social participation and political efficacy in rural areas. The item in the Social Participation Survey is “Have you participated in the following over the past 2 years?” The participation options are provided as follows: reflecting on social issues in newspapers, radio, online forums, and other media or government departments; participating in village (residential)committee elections; participating in significant decision-making discussions in the village/unit; and participating in online/offline collective action for the maintenance of rights. The assigned value for participation is 1. Political efficacy is operationalized into two aspects: perception of political comment ability and degree of political concern. The related question in the questionnaire is, “Do you agree with the following statement?” The following statements are given: (1) People’s participation in political activities is useless and has no fundamental impact on the government; (2) People should listen to the government; (3) Subordinates should listen to their superiors. (4) My freedom of speech is restricted by the government; and (5) The government governs state affairs, and the ordinary people do not need to consider my ability and knowledge too much to comment on politics and participate in political activities. The degree of consent is measured from high to low as 1–5 points, such that the higher the score, the stronger the political efficacy is. In this study, factor analysis was conducted on these four subjects. Through Bartlett's spherical test, principal component analysis extracted a factor according to the factor load, the political efficacy factor. The KMO value was 0.645, and the cumulative variance contribution rate was 46.970%. The *p*-value of the Butterritsphericity test was < 0.001.

#### Control variables

Following previous studies, this study takes gender, age, marital status, education level, and number of children as control variables. Considering the possible quadratic effect of age, we use the square of age as a variable and the control variable of age. Marital status is a dummy variable (0 = unmarried, 1 = married). We uniformly define unmarried, divorced, widowed, and unclear status as unmarried and define first marriage with the spouse, remarriage with a spouse, and cohabitation as married.

The basic statistics for the model variables are shown in Table 2.

**Table 2:** Descriptive statistics of variables.

Variable type	Variable name	Sample size	Mean value	Minimum value	Maximum value	Standard deviation
Dependent variables	Life satisfaction	1552	7.24	1	10	2.43
Control variables	Gender (male = 1)	1552	1.53	1	2	0.49
	Age	1552	64.53	60	69	2.72
	Marital status (married = 1)	1552	0.85	0	1	0.35
	Education level	1552	2.21	1	12	1.16
	Number of children	1527	1.31	0	5	0.80
Independent variables						
Socioeconomic security	Total household income	1507	42,639.05	11	2,005,500	84,838.96
	Housing security	1486	1.27	1	6	0.546
	Pension insurance participation	1551	0.73	0	1	0.43
	Public safety perception	1552	4.75	2	10	1.44
Social cohesion	Social groups and market trust factor	1552	0	− 3.68	3.15	1
	Central governmentand institutional trust factor	1552	0	− 5.02	3.07	1
	Local government and general trust factor	1552	0	− 3.45	2.57	1
	Evaluation of the legal level of social morality	1552	14.48	2	20	3.86
	Social identity factor	1552	0	− 5.60	.99	1
Social inclusion	Evaluation of social tolerance	1552	7.12	1	10	2.34
	Evaluation of social equity	1552	7.06	1	10	2.47
	Public service perception factor	1552	0	− 3.24	1.55	1
Social empowerment	Social participation	1552	0.73	0	5	0.87
	Political efficacy factor	1552	0	− 1.62	3.29	1

## Statistical analysis and findings

Because the dependent variable is life satisfaction, a continuous variable, this study uses multiple linear regression to identify the influence of social quality factors on the life satisfaction of older adults in rural areas. To explore the correlation between rural social quality and life satisfaction among older adults in rural areas, based on the control of gender, age, marital status, education level, number of children, and other related variables, four conditional factors of social quality were included. The socioeconomic security and social cohesion, inclusion, and empowerment model (models 2–5) and the joint model of four conditional factors of social quality (model 6) were established. The specific statistical results are shown in Table 3.

**Table 3:** Linear regression model of social quality affecting the life satisfaction of the elderly.

Variable	Model1	Model 2	Model 3	Model 4	Model 5	Model 6
Control variables						
Gender (male = 1)	− 0.091	0.058	− 0.107	0.014	− 0.030	0.149
	(0.134)	(0.137)	(.129)	(0.124)	(0.136)	(0.129)
Age	− 0.253	− 0.260	0.601	0.733	0.220	0.137
	(1.165)	(1.191)	(1.109)	(1.073)	(1.164)	(1.095)
Marital status (married = 1)	− 0.002	0.002	− 0.004	− 0.005	− 0.001	− 0.001
	(0.009)	(0.009)	(0.009)	(0.008)	(0.009)	(0.008)
Education level	0.332*	0.275	0.356**	0.337**	0.335*	0.326*
	(0.181)	(0.185)	(0.173)	(0.167)	(0.181)	(0.171)
Number of children	0.074	0.065	0.086	0.080	0.063	0.060
	(0.057)	(0.058)	(0.055)	(0.053)	(0.058)	(0.054)
Gender (male = 1)	− 0.166**	− 0.208***	− 0.214***	− 0.173**	− 0.168**	− 0.216***
	(0.078)	(0.079)	(0.074)	(0.072)	(0.078)	(0.073)
Independent variables						
Socioeconomic security						
Total household income		0.235**				0.117
						(0.099)
		(0.107)				
Housing security		0.333***				0.304***
						(0.107)
		(0.117)				
Pension insurance participation		0.524****				0.326**
						(0.138)
		(0.149)				
Public safety perception		− 0.014				0.088**
Social cohesion						(0.042)
		(0.044)				
Social groups and market trust factor			− 0.164***			− 0.148**
						(0.060)
			(0.060)			
Central government and institutional trust factor			0.004			− 0.006
						(0.060)
			(0.060)			
Local government and general trust factor			0.389****			0.118*
						(0.068)
			(0.064)			
Evaluation of the legal level of social morality			0.119****			0.054***
						(0.019)
			(0.017)			
Social identity factor			0.136**			− 0.031
						(0.067)
			(0.064)			
Social inclusion						
Evaluation of social tolerance				0.163****		0.131****
						(0.029)
				(0.028)		
Evaluation of social equity				0.214****		0.164****
						(0.030)
				(0.028)		
Public service perception factor				0.321****		0.314****
						(0.073)
Social empowerment						
				(0.063)		
Social participation					0.177**	0.129*
					(0.073)	(0.069)
Political efficacy factor					− 0.025	0.102
					(0.062)	(0.063)
Constant	− 2.029	12.725	− 14.886	− 20.061	− 1.173	− 2.866
	(37.533)	(38.375)	(35.716)	(34.576)	(37.509)	(35.285)
N	1526	1426	1526	1526	1526	1426
F	2.052*	3.978****	16.407****	32.294****	2.310**	16.235****
AdjustedR^2^	0.012	0.020	0.100	0.156	0.007	0.176

Standard error in parentheses.

*p < 0.1; **p < 0.05;***p < 0.01; ****p < 0.001.

According to the six regression models in Table 3, this study produces the following findings. Model 1 is a benchmark model that includes only control variables. The results indicate no significant differences in gender, age, and education level in the life satisfaction of older adults in rural areas. Still, there are differences in marital status and the number of children. Specifically, the life satisfaction of the married elderly in rural areas is higher than that of the unmarried elderly in rural areas, which may reflect the positive role of marriage. The number of children is negatively correlated with the life satisfaction of older adults in rural areas, which may be for the following two reasons: because of the inflow of rural labor force into cities, the function of rural family education and pensions are gradually weakened. On the one hand, this increases the burden of grandchild care for older adults in rural areas. On the other hand, the pension problems of older adults in rural regions face various challenges, such that the needs of older adults in rural areas cannot be met.

This study focuses on the impact of rural social quality factors on the life satisfaction of older adults in rural areas. Of the six models established, model 6 shows the best fit. That is, after incorporating the four dimensions of social quality. However, the influence of some dimensions of social quality and some related indicators on the life satisfaction of older adults in rural areas people is weaker, such as social identity in the dimension of social cohesion, the influence of public safety perception on the dimension of social and economic security has changed, becoming obvious from having been unobvious. The model fitting degree of social quality showed that it affected the life satisfaction of older adults in rural areas, which increased it by 16.4–17.6%, which may indicate that the four conditional factors of social quality are essential factors that affect the life satisfaction of older adults in rural areas.

From the impact of control variables, gender, age, and education level still have no significant impact on the life satisfaction of older adults in rural areas. However, marital status and the number of children have substantial indigenous effects. The impact of marital status on the life satisfaction of older adults in rural areas was significant at *p* < 0.1, and the life satisfaction of the married elderly in rural areas is higher than that of the unmarried ones. The effect of the number of children on life satisfaction was significant at *p* < 0.01. The coefficient is still negative, which is inconsistent with the findings of previous studies^35^, indicating that compared with older adults in rural areas, people with more women and elderly in rural areas people with fewer children have higher life satisfaction. One important reason may be that high-intensity grandparent care reduces the life satisfaction of older adults in rural areas^9^, consistent with previous studies^36^.

### Socioeconomic security and life satisfaction of elderly

Unlike the case of model 2, which only includes the dimension of socioeconomic security, the results for model 6 indicate that after the four conditional factors of social quality are included, the influence of the Variable of family income on the life satisfaction of older adults in rural areas in the socioeconomic security factor was no longer significant, but the impact of housing conditions and pension insurance participation variables remains substantial; in particular, the influence of the Variable of public safety perception is changed from not obvious to obvious. This shows that the influence of the life satisfaction of older adults in rural areas is tied to not only the absolute income of the family but also the comprehensive perception of people's economic conditions. Older adults in rural areas pay more attention to comparative experience from relative poverty. Hypothesis 1 is partially confirmed.

Compared with model 2, which only includes the dimension of socioeconomic security, model 6 shows that after the four conditional factors of social quality are included, the influence of family income variables on the life satisfaction of older adults in rural areas in social and economic security factors is no longer significant. However, the influence of housing conditions and endowment insurance participation variables is still significant, especially with respect to the influence of public safety perception variables, which changes from not significant to significant, showing that the influence of elderly in rural areas life satisfaction concerns not only the absolute income of the family but also the comprehensive perception of residents' economic status. A more reasonable explanation is the comparative experience of relative poverty, which older adults in rural areas are more concerned with. Hypothesis 1 is partially confirmed.

Absolute family income is not statistically significant. Housing conditions, pensions, and public security are more influential than absolute family income. This is because the absolute family income considers economic burden, consumption expenditure, and other factors. Good housing conditions and pension security mean that the life of older adults in rural areas can still be maintained at a decent level after expenses have been deducted^22^, which provides psychological support, strengthening older adults in rural areas’ sense of adequacy in life.

The high quality of living among older adults in rural areas is based on a steadily improved social security policy framework. Housing conditions and old-age security are essential material foundations for increasing the life satisfaction of older adults in rural areas. The elderly in rural areas experience increased pension demands as the rural family pension function deteriorates. Old-age security is important to older adults in rural areas’ fundamental living and health security. Greater availability of health and old-age security in rural regions would considerably increase happiness among older adults in rural areas. Currently, the requirements for this group are steadily evolving from eradicating poverty and obtaining food and clothes to obtaining essential quality of life. The continual improvement of residential circumstances and old-age security gives a more satisfying material life quality experience to fulfill the living demands of older adults in rural areas, which may considerably increase rural seniors' life satisfaction. For example, for each degree of housing condition, the life satisfaction of older adults in rural areas rises by 0.304.

Public safety perceptions influence older adults' happiness in rural areas. Public safety is a pleasant psychological experience for older adults in rural areas to seek high-quality living, and it is the material basis for enhancing their life satisfaction. The impression of public safety among older adults in rural areas improved by one unit, and their life satisfaction improved by 0.088 points. However, hidden threats in rural society, including unlawful and criminal activities and food and drug safety, considerably impair the sense of environmental security among the elderly in rural areas, lowering their level of happiness.

### Elderly in rural areas social cohesiveness and life satisfaction

Regarding the influence of social cohesiveness on rural senior life satisfaction, the statistical findings of the model6 suggest that the degree of social morality and legal system had a favorable role in increasing the life satisfaction of rural older adults. Each unit of improvement in the rating of social morality and the legal system enhances the life satisfaction of older adults in rural areas by 0.054 points. This demonstrates that an excellent social, moral, and legal environment, a central core of life happiness, provides the foundation for rural older adults to obtain a better life. Conversely, if older adults in rural communities are regularly exposed to unlawful or harmful moral acts, it indicates that societal value standards are not effectively recognized in rural regions. This will undoubtedly impair the capacity to foresee others' conduct among older adults in rural areas, limiting their potential to increase their life pleasure. The three social trust variables (social group and market trust factor, central government and institutional trust factor, and local government and general trust factor) have varying impacts on rural senior life satisfaction.

Social groups, market trust elements, local government, and general trust factors substantially influence life satisfaction among older adults in rural areas. The central government and institutional trust factors were not significant. The determinants of local governance and general trust substantially influence life satisfaction among older adults in rural areas (*p* < 0.1), and the regression coefficient is positive. That is an increase in faith in local government and generally dramatically improves the living satisfaction of older adults in rural areas. The life contentment of older adults in rural areas rises by 0.118 points for every unit increase in local government and general trust, demonstrating that social capital may actively improve the life satisfaction of older adults in rural areas. Community and market trust was statistically significant (*p* < 0.05), although the regression coefficients were negative. Life satisfaction among older adults in rural areas is inversely associated with community and market trust. Hypothesis 2 was partically confirmed.

### Social inclusion and life satisfaction of the elderly in rural areas

The results show that the three indicators of social inclusion, tolerance, and equity and public service perception passed the statistical significance test (*p* < 0.001), indicating that the degree of social tolerance, social equity, and public service provided by the government significantly improved the life satisfaction experience of elderly residents in rural areas. The results show that for every unit increase in social tolerance, the life satisfaction of older adults in rural areas increases by 0.131 points. The life satisfaction of older adults in rural areas increased by 0.164 points per unit of change in social equity evaluation. The life satisfaction of older adults in rural areas increased by 0.314 points per unit of public service promotion. Hypothesis 3 is validated.

### Social empowerment and life satisfaction of rural elderly

Model 6 showed that social participation was statistically significant (*p* < 0.1). Every one-unit increase in social participation was correlated with life satisfaction among older adults in rural areas, an increase of 0.129 points. Social participation can enhance social interaction, help obtain emotional resources and accumulate social capital to expand social support networks among older adults in rural areas and improve their life satisfaction. However, the impact of political efficacy perception on older adults in rural areas' life satisfaction is not statistically significant. This may be because older adults in rural areas still take survival security as the core goal in meeting basic life needs, medical care, and pension. The influence of political efficacy perception on the life experience of older adults in rural areas is relatively small. Based on available data, Hypothesis 4 is thus partially confirmed.

Most indicators for the four conditional factors of social quality are highly relevant to the life satisfaction of older adults in rural areas. The statistical results show that the life satisfaction of older adults in rural areas is not simply a subjective state but largely based on rural social development and sharing the development achievements with older adults in rural areas. Rural socioeconomic security and social cohesion, inclusion, and empowerment have improved life satisfaction in four aspects: material security, institutional basis, psychological experience, and power basis. Socioeconomic security was related to pension security, and other factors have a decisive impact on enhancing the life satisfaction of older adults in rural areas. Strengthening social and livelihood security in rural areas and promoting actual access to older adults in rural areas are among the most critical goals for rural revitalization. Improvements in life satisfaction stem from the satisfaction of the material and spiritual needs of members of society. However, for older adults, the satisfaction of material life security is still the priority in the demand sequence. Spiritual needs can be considered more closely only when the material living standard is improved to a certain extent. Acontinuouslyimprovingsocial security system will be able proactively to respond to the demands of older adults, providing space bottom line and policy support for a better life for senior residents.

## Discussion and conclusions

The life satisfaction of older adults in rural areas was a manifestation of their well-being and an important entry point for testing the quality of rural social development in China. The control variables of marital status and number of children were shown to impact life satisfaction. Overall, the four conditional factors of social quality are highly correlated with the life satisfaction of older adults in rural areas. Additionally, the housing conditions, pension insurance participation, social groups, market trust factors, local government and general trust factors, social participation, and other independent variables were found to have significant effects on the life satisfaction of older adults in rural areas, especially for social tolerance, social equity, and public service perception that fall under the dimension of social inclusion and still pass the statistical test. The influence of particular variables is not apparent everywhere in the model, precisely household income under the dimension of social, economic security, trust, and social identity between the central government and institutions about the dimension of social cohesion, as well as the political efficacy factor, about the dimension of social empowerment.

Constant improvements to the social security system can eliminate the worries regarding health and pension problems among older adults in rural areas, promote a better life among the elderly in rural areas, and provide better experiences. This indicates that over a long period, the demand for older adults in rural areas is still dominated by the improvement of social security and, more importantly, the progress of old-age security, significantly alleviating relative poverty. China has entered a post-poverty era. The task of poverty alleviation has shifted from absolutely solving poverty to reducing or alleviating relative poverty. The key to rural revitalization is to enable rural residents to share the fruits of rural economic and social development by alleviating relative poverty to improve the social quality of rural areas in China. Therefore, while improving the absolute material living standard, it is necessary to build a long-term mechanism to alleviate relative poverty to improve the life satisfaction of older adults in rural areas in China.

Rural social cohesion is an important institutional basis for generating a sense of good life among older adults in rural areas. In terms of social trust, social groups, and market trust factors, local government and general trust factors significantly impact life satisfaction among elderly in rural areas people^28^. A primary difference between this study and previous studies can be found in that, for example, the life satisfaction of older adults in rural areas is negatively correlated with community and market trust here. This seems to mean that, in context and explicitly the crisis "of confidence" era, elderly in rural areas people face more risk of being cheated, significantly increasing their social interaction and transaction costs and weakening their life satisfaction experience. Local government and interpersonal trust positively impact the life satisfaction of older adults in rural areas. As local government and interpersonal trust measures improve, the life satisfaction level of older adults in rural areas gradually increases. This shows that the institutional commitment of the local government conforms to normative expectations of older adults in rural areas. The local government provides relevant resources and policy support to achieve a better life for older adults in rural areas so that they can benefit from the achievements of poverty alleviation and improve their life satisfaction.

Good social inclusion is the psychological experience of older adults in rural areas and life satisfaction. Social tolerance and equity evaluation, perception, and public services in social inclusion positively affect rural older adults' life satisfaction. The inclusive characteristics of the poverty alleviation policy provide an institutional environment for reducing rural social barriers and exclusion. When older adults in rural areas perceive a fair and just social environment and good public services, they consider that their needs are responded to and met promptly and their multiple demands are respected. They are driving the generation of a sense of good life experience for older adults in rural areas, and their life satisfaction is also significantly improved.

First, social tolerance is the evaluation of the social acceptance of different groups. In recent years, with the deepening of social differentiation in China, the sense of relative deprivation caused by social inequality has dramatically weakened the positive experience brought about by improving material living standards^27^. Higher social inclusion and tremendous respect for the rights of older adults in rural areas, together with reducing the sense of exclusion experienced by older adults in rural areas, are the essential psychological bases for strengthening older adults in rural areas to obtain better life experiences. On the contrary, social exclusion and discrimination are not conducive to improving life satisfaction. Higher social tolerance has a positive role in promoting the generation of older adults in rural areas' sense of good life experience. Elderly in rural regions in society with higher tolerance exhibit higher life satisfaction. As the in-depth promotion of the rural revitalization strategy proceeds, the living standard of older adults in rural areas has gradually improved. Simultaneously, some new values and things continue to emerge. If older adults in rural areas have a high degree of tolerance for these new concepts and new things, it will help them with their life experiences. Second, social justice cognition shows the psychological induction effect on improving the life satisfaction of older adults in rural areas. The aggravation of social injustice will affect people's daily life mood and social judgment. The social environment can affect the current sense of no life experience of older adults in rural areas. It should become one of the essential focuses of local governance in rural areas. Finally, public services provide a central basis for forming a positive psychological experience to improve the life satisfaction of older adults in rural areas. In the process of rural revitalization, if the government makes policies in response to the needs of older adults in rural areas, responds timely to their diversified demands, and provides professional services so that older adults in rural areas people benefit from it, a good policy will significantly enhance their life satisfaction. The above analysis shows that life satisfaction cannot be separated from a specific social context. It goes beyond a subjective feeling to members of society who are gradually being generated in the sharing process of social development achievements.

Social empowerment is an important functional basis affecting life satisfaction among rural older adults. Social participation in social empowerment has a positive role in promoting the life satisfaction of this group. Social participation may enhance social interaction and help older adults in rural areas to obtain emotional resources, accumulate social capital, and expand their social support network, thereby improving and strengthening the life satisfaction of older adults in rural areas. However, the impact of political efficacy perception on older adults in rural areas' life satisfaction is not statistically significant. The possible reason is that older adults in rural areas, at present, still take survival security as the core goal to meet the needs of basic life, medical care, and pension. The influence of the perception of political efficacy on the life experience of older adults in rural areas is relatively tiny. The possible reason lies in the reality of the lack of political participation ability of older adults in rural areas in China. Due to the influence of education level and urban–rural dual structure, the political participation of older adults in rural areas is still relatively insufficient.

Social quality is not only a purely instrumental concept. It has a strong policy practice orientation^22^^.^ The starting point of the social quality theory is to seek to resolve the contradiction between social and individual development to improve the social situation^21^. Therefore, the purpose of the study is not only to describe the impact of various factors of social quality on society but also to improve social quality, using social quality indicators for social governance^20^. The study of the quality of rural society in China helps provide a reference for formulating rural social governance policies. Quality Social quality theory indicates that we should pay attention to the value concept of collective welfare and individual empowerment. The enlightenment is that in a high-quality society, we should coordinate the relationship between social members and social development, pay attention to the well-being of older adults in rural areas, and respect the appeals of the elderly in rural areas and their sense of life experience.

Regarding rural revitalization and governance innovation in rural social governance, it is the core value goal to actively respond to the basic national conditions of China’s aging population, actively seek out ways of exploring the path to comprehensively improve the quality of rural society, improve the relative poverty in rural areas, and continue to improve the social and economic security of elderly in rural areas people. Simultaneously, it is also rural revitalization’s core value pursuit to comprehensively combine the “medium- and long-term plan of the state to respond to the aging of the population actively”; build an elderly-friendly society; form a good atmosphere for the participation of older adults, families, society, and the government; achieve social cohesion; enhance social inclusion; and achieve social empowerment, thereby improving the life satisfaction of elderly in rural areas.

By examining the influence of social quality factors on the life satisfaction of rural elderly populations, our research opens up a new avenue for analyzing the determinants of life satisfaction in these communities. This approach not only broadens the scope of research related to life satisfaction but also enriches the theoretical framework of social quality. Furthermore, it provides a robust basis for applying social quality theory in policy-making, potentially guiding interventions aimed at enhancing the well-being of rural elderly individuals.

Our study represents a significant advancement over traditional research in this field, which typically examines the effects of isolated factors on elderly life satisfaction. Instead of focusing solely on individual variables, our work considers the comprehensive social environment of the elderly. We provide a systematic analysis of how social quality factors collectively influence elderly life satisfaction at both individual and societal levels. This holistic approach not only deepens our understanding but also highlights areas for potential intervention.

This study not only offers a comprehensive theoretical and empirical examination of how social quality impacts the life satisfaction of the rural elderly in China, but it also establishes a strong empirical foundation for the practical application of these theories within the Chinese context. Furthermore, the findings provide valuable insights that can inform the theoretical expansion of rural social governance. Our theoretical framework and empirical results equip governmental bodies with evidence-based guidance that can be utilized to craft social policies aimed at enhancing the life satisfaction of elderly populations in rural areas.

Future research should continue to explore and expand upon the social quality system to develop targeted strategies for enhancing life satisfaction among the elderly. This entails not only a deeper investigation into the existing dimensions of social quality but also identifying and integrating new factors that influence elderly well-being. Such studies will be crucial for creating more effective interventions and policies that can significantly improve the quality of life for older adults.

### Supplementary Information

Supplementary Information.

## Data Availability

The data was drawn from CSS2019, a large-scale nationwide public survey conducted by the Institute of Sociology of the Chinese Academy of Social Sciences (http://csqr.cass.cn/). The data can be utilized after an application has been submitted and approved by the Institute of Sociology of the Chinese Academy of Social Sciences.

## References

[1] Jin L (2011). The influence factors of life satisfaction for elderly and their comparative analysis. Popul. Econ..

[2] Ng S, Ng YK (2001). Welfare-reducing growth despite individual and government opitimization. Soc. Choice Welf..

[3] Xia YF (2014). Enlightenment of social quality theory on social policy construction in China. Soc. Sci. Hunan.

[4] Midgley J, Miao ZM (2009). Social Development: The Developmental Perspective in Social Welfare.

[5] Parra-Rizo MA, Vásquez-Gómez J, Álvarez C, Diaz-Martínez X, Troncoso C, Leiva-Ordoñez AM, Zapata-Lamana R, Cigarroa I (2022). Predictors of the level of physical activity in physically active older people. Behav. Sci..

[6] Zapata-Lamana R, Poblete-Valderrama F, Ledezma-Dames A, Pavón-León P, Leiva AM, Fuentes-Alvarez MT, Cigarroa I, Parra-Rizo MA (2022). Health, functional ability, and environmental quality as predictors of life satisfaction in physically active older adults. Soc. Sci..

[7] Parra-Rizo MA (2017). Most valued components of the quality of life in older people than 60 years physically active. Eur. J. Investig. Health Psychol. Educ..

[8] Yuan H, Ma D (2011). Subjective well-being from the perspective of social quality: An empirical study based on Shanghai. Jilin Univ. J. Soc. Sci. Ed..

[9] Wang P, Li SZ (2011). A longitudinal study of the dynamic effect of intergenerational support on life satisfaction of rural elderly. Popul. Res..

[10] Diener E, Suh EM, Lucas RE, Smith HL (1999). Subjective well-being: Three decades of progress. Psychol. Bull..

[11] Helliwell JF, Putnam RD (2004). The social context of well-being. Philos. Trans. R. Soc. B Biol. Sci..

[12] Hu R, Ye LY (2014). Subjective socioeconomic status and class identity of urban residents. Soc. Sci. Heilongjiang.

[13] Ma D (2015). The influence of social network on life satisfaction: A two-wave longitudinal study of Beijing, Shanghai and Guangdong. Chin. J. Sociol..

[14] Xiang YH, Yao H (2017). A research on the life satisfaction of rural elderly in China. J. Stat. Inf..

[15] Song LN, Appleton S, Xiao H (2014). Life satisfaction in urban areas of China: Elements and determinants. Foreign Theor. Trends.

[16] Wang JX (2013). Social services: The experience and enlightenment of Hong Kong. J. Gansu Administration Inst..

[17] Shen, R. Y. & Zhao, Z. Y. Research on the influencing factors of urban residents’ subjective satisfaction—Based on the survey data of Beijing, Tianjin and Shanghai. *World Surv. Rev.* **8**, 50–55 (2015).

[18] Beck W, Maesen L, Walker A (1997). The Social Quality of Europe.

[19] Beck W (2001). Social Quality: A Vision for Europe.

[20] Lin K (2010). Social quality theory: A new perspective for the studies of harmonious society. J. Renmin Univ. China.

[21] Gao, H. Z. On the Construction of Rural People’s Livelihood from the Perspective of Social Quality: An Analysis Based on 2013CSS Rural Sample. *Population and Society* **33**(01), 52–62 (2017).

[22] Zhang HD (2010). From development road to social quality: Paradigm shift of social development research. Jianghai Acad. J..

[23] Nie W, Cai PP (2021). Making cities more friendly to youth development: The impact of social quality on youth’s sense of gain. China Youth Study.

[24] Xu YH, Li ZB (2021). Social quality and urban residents’ sense of gain. Nankai J..

[25] Wang P, Li SZ (2011). A longitudinal study of the dynamic effect of intergenerational suppport on life satisfaction of rural elderly. Popul. Res..

[26] Abbott P, Wallace C, Claire W (2009). Comparing Social Quality 27 EU Countries in Time: 2003 and 2007. Social Quality in Work and Care.

[27] Abbott P, Wallace C (2012). Social quality: A way to measure the quality of society. Soc. Indic. Res..

[28] Yang MH (2015). Research on Chinese social quality index system based on social harmony construction. J. Southwest Minzu Univ..

[29] He LX, Pan CY (2011). Uncover China's "Easterlin Paradox": Income gap, uneven opportunities and happiness. J. Manag. World.

[30] Maesen L, Walker AC (2005). Indicators of social quality: Outcomes of the European scientific network. Eur. J. Soc. Qual..

[31] Maesen L, Walker A (2012). Social Quality: From Theory to Indicators.

[32] Zhu M (2023). The effect of political participation of Chinese citizens on government satisfaction: Based on modified causal forest. Proc. Comput. Sci..

[33] Zhang J, Gong X, Zhu Z, Zhang Z (2023). Trust cost of environmental risk to government: The impact of internet use. Environ. Dev. Sustain..

[34] Li Y, Guan H, Fu H (2023). Understanding financial risk protection in China’s health system: A descriptive analysis using data from multiple national household surveys. BMC Public Health.

[35] Wang, X. H. & Xia, K. K. Factors Affecting Life Satisfaction and Depression of Rural Elderly. *Social Welfare*, **9**, 55-61(2021)

[36] Yang, X. T. & Bi, H. X. The influence of intergenerational support on life satisfaction of the rural elderly: Based on CHARLS data. *Scientific Res. Aging* **9**(04), 13–24 (2021).

