# Supplementary material for: A nationwide study of the impact of social quality factors on life satisfaction among older adults in rural China
Source: Sci Rep. 2024 May 21;14:11614. doi: 10.1038/s41598-024-61398-4 (PMC11109087; doi:10.1038/s41598-024-61398-4)
Supplement: Supplementary file 1 — Supplementary Information. [file 41598_2024_61398_MOESM1_ESM.pdf]

2019 Comprehensive Survey of China's Social Conditions

（CSS 2019）

Questionnaire

CSS.CSSN.CN

Institute of Sociology, Chinese Academy of Social Sciences

June 2019

Dear Sir/Madam!

My name is____, Yes ___________________Visitor of the University/Academy of Social Sciences.We are conducting a social survey to understand people's employment, work and living conditions, as well as their views on some current social problems.We have selected your family as the access object.Now I want to know about your family members. Thank you for your support.

[illegible]

2019 Comprehensive Survey of China's Social Conditions

1. Questionnaire No.:[__|_____|_____|_____|_____|_____]

2. Sample No.:[_______|_____|_____|_____|_____|_____]

3. CAPI auto load access location:

State:____

City+County/District Name:______________________________

4. Is the respondent: 1. Yes 2. No

5. Visitor (signature)_______________ Code:[| | |]

6. Accompanying supervisor (signature)____________________ Code:[| | |] **Patrol supervision (signature)______________________ Code:**[| | |]

7. Review (signature)__________ Code:[| | |] **Patrol supervision (signature)_ Code:** [| | |]

8. On site review type: 1. In house review 2. Telephone review 3. Accompanying review 4. Not rechecked

Review (signature)_____________Code:[| | |] **Patrol supervision (signature)__ Code:**[| | |]

9. Access start time: [_|__________]At [_ | _] on [_ | _][_______________|________]Minute (24-hour);End time:

Day [_ | _][____________|_____]Hour[____|_____]Minutes (24-hour system)

10. Total access length:[____|______|______](minutes)

Article 25 of the Statistics Law of the People's Republic of China stipulates that any unit or individual shall not provide, disclose or use for purposes other than statistics the information obtained in statistical investigation that can identify or infer the identity of a single statistical investigation object.

The following visit officially begins

Dear Sir/Madam!

My name is______, is a visiting member of the Chinese Academy of Social Sciences.We are conducting a social survey to understand people's employment, work and living conditions, as well as their views on some current social problems.After strict scientific sampling, we selected you as the survey object.Your cooperation is of great significance for us to understand relevant information and formulate social policies.

There is no right or wrong answer to the questions in the questionnaire. You only need to answer

Part A: Residents

<u>**First of all, I'd like to know some basic information about you and your family for analysis only. I hope you don't mind**</u>

A1a. First of all, please tell me how many people are in your family?What is their relationship with you? **Record:[________|_______]Oral person**

a. Relationship with respondents	b. Gender	c. Year of birth	d. Education:	e. Marital status:	f. The current working conditions are:	G1. Does he/she currently eat and live with you:	G2. Does he/she currently share income and expenditure with you:
01. husband<br>02. wife<br>03. Son<br>04. daughter<br>05. father<br>06. mother<br>07. Spouse father<br>08. Spouse mother<br>09. grandfather<br>10. grandmother<br>11. Grandfather<br>12. Grandmother<br>13. Daughter-in-law<br>14. son-in-law<br>15. grandson<br>16. granddaughter<br>17. Grandson's wife<br>18. Granddaughter's husband<br>19. Grandchild<br>20. daughter's daughter<br>21. Wife of my grandson<br>22. Granddaughter's husband<br>23. brother<br>24. sisters<br>25. Brother's wife<br>26. Sister's husband<br>27. Other relatives			[Show the card on page 1]<br>01. Not attending school<br>02. primary school<br>03. junior middle school<br>04. high school<br>05. secondary specialized school<br>06. Vocational school<br>07. College<br>08. undergraduate college<br>09. graduate student<br>10. Others (please specify:____)<br>98. [Unclear]	[Show the card on page 2]<br>1. unmarried<br>2. First marriage with spouse<br>3. Remarriage with spouse<br>4. divorce<br>5. Widow<br>6. cohabitation<br>8. [Unclear]	[Show the card on page 3]<br>1. Only farming<br>2. Only engaged in non-agricultural work<br>3. Mainly engaged in non-agricultural work, but also engaged in agriculture<br>4. Mainly engaged in agriculture, but also engaged in non-agricultural work<br>5. be unemployed<br>6. retire<br>7. Preschool children or schoolchildren<br>8. Others (please specify)<br>98. [Unclear]	1. Eat and live together<br>2. Live together, but not eat together<br>3. Eat together, but not live together<br>4. We don't eat and live together	**1. Both income and expenditure are together**<br>2. Inconsistency between income and expenditure
Respondent	Male<br>Female	___ ___ ___ ___ Year<br>___ ___ Month					
		___ ___ ___ ___ Year					
		___ ___ ___ ___ Year					
		___ ___ ___ ___ Year					
		___ ___ ___ ___ Year					
		___ ___ ___ ___ Year					
		___ ___ ___ ___ Year					
		___ ___ ___ ___ Year					
		___ ___ ___ ___ Year					
		___ ___ ___ ___ Year					
		___ ___ ___ ___ Year					
		___ ___ ___ ___ Year					

a. Relationship with respondents	b. Gender	c. Year of birth	d. Education:	e. Marital status:	f. The current working conditions are:	G1. Does he/she currently eat and live with you:	G2. Does he/she currently share income and expenditure with you: 1. Both income and expenditure are together
01. husband<br>02. wife<br>03. Son<br>04. daughter<br>05. father<br>06. mother<br>07. Spouse father<br>08. Spouse mother<br>09. grandfather<br>10. grandmother<br>11. Grandfather<br>12. Grandmother<br>13. Daughter-in-law<br>14. son-in-law<br>15. grandson<br>16. granddaughter<br>17. Grandson's wife<br>18. Granddaughter's husband<br>19. Grandchild<br>20. daughter's daughter<br>21. Wife of my grandson<br>22. Granddaughter's husband<br>23. brother<br>24. sisters<br>25. Brother's wife<br>26. Sister's husband<br>27. Other relatives			[Show the card on page 1]<br>01. Not attending school<br>02. primary school<br>03. junior middle school<br>04. high school<br>05. secondary specialized school<br>06. Vocational school<br>07. College<br>08. undergraduate college<br>09. graduate student<br>10. Others (please specify:____)<br>98. [Unclear]	[Show the card on page 2]<br>1. unmarried<br>2. First marriage with spouse<br>3. Remarriage with spouse<br>4. divorce<br>5. Widow<br>6. cohabitation<br>8. [Unclear]	[Show the card on page 3]<br>1. Only farming<br>2. Only engaged in non-agricultural work<br>3. Mainly engaged in non-agricultural work, but also engaged in agriculture<br>4. Mainly engaged in agriculture, but also engaged in non-agricultural work<br>5. be unemployed<br>6. retire<br>7. Preschool children or schoolchildren<br>8. Others (please specify)<br>98. [Unclear]	1. Eat and live together<br>2. Live together, but not eat together<br>3. Eat together, but not live together<br>4. We don't eat and live together	2. Inconsistency between income and expenditure
		___ ___ ___ ___ Year					
		___ ___ ___ ___ Year					
		___ ___ ___ ___ Year					
		___ ___ ___ ___ Year					
		___ ___ ___ ___ Year					
		___ ___ ___ ___ Year					
		___ ___ ___ ___ Year					
		___ ___ ___ ___ Year					
		___ ___ ___ ___ Year					
		___ ___ ___ ___ Year					
		___ ___ ___ ___ Year					
		___ ___ ___ ___ Year					
		___ ___ ___ ___ Year					
		___ ___ ___ ___ Year					
		___ ___ ___ ___ Year					

[Interviewer's question] In addition to the people just mentioned, have any members of your family gone out or not lived in this house (such as students studying outside and family members working for part-time jobs)?If yes, please tell us some simple information about them.[If yes, CAPI prompts the interviewers to increase the "family population"]

A1b. Among the family members just now________Who is the husband wife relationship?

	Member 2	Member 3	Member 4	Member 5	Member 6
Member 1					
Member 2					
Member 3					
Member 4					
Member 5					

A1c.[Note: 1. If the following four respondents did not answer the question A1a, CAPI needs to load the corresponding person in the following table; if there is corresponding answer in question A1a, it does not need to be loaded in the following table; 2. If the respondent is unmarried, it does not need to load the spouse and spouse's parents]

Ask your parents and spouse's parents some simple information: [Note to interviewer: there is no jumping answer to this question]

a. Relationship with respondents:<br>(Note: Gender Capi automatically gives value): 1. Male<br>2. Female	b. Year of birth:<br>Unified numbering[Unclear]	c. Education:<br>[Show card Page 1]<br>01. Not attending school<br>02. primary school<br>03. junior middle school<br>04. High School<br>05. Technical secondary school<br>06. Vocational school<br>07. College<br>08. Undergraduate<br>09. Postgraduates<br>10. Others (please say Ming:_____)<br>*98. [Unclear]*	d. Current or last employment status:<br>[Show the card on page 4]<br>01. Agriculture only<br>02. Only engaged in non-agricultural work<br>03. Mainly engaged in non-agricultural work, but also Farming<br>04. Mainly engaged in agriculture, but also engaged in non agriculture work<br>05. Unemployed<br>06. Retirement<br>07. Others (please specify)<br>*98. [Unclear]*	e. Is he alive<br>1. yes<br>2. no<br>*8. [Unclear]*<br>*9. [No answer]*
1. Respondent's father	[___|__ ||]Year	[___|__]	[___|__]	[__]
2. Respondent's mother	[___|__ ||]Year	[___|__]	[___|__]	[__]
3. Spouse father	[___|__ ||]Year	[___|__]	[___|__]	[__]
4. Spouse mother	[___|__ ||]Year	[___|__]	[___|__]	[__]

A2. Your nationality is: (single choice)

Han nationality.. 1
Mongolian.. 2
Manchu.. 3
Hui nationality.. 4
the zang or tibetan people.. 5
Zhuang nationality.. 6
Uygur ethnic group.. 7
Others (please specify).. 8
unclear.. 98

A3. Your political outlook is: (single choice)

CPC member 1
communist youth league member 2
Democratic parties 3
The masses 4

A4a. Your current account nature is: (single choice)

Agricultural household registration 1
Non agricultural household registration 2
Others (please specify): 3

A4b. Your current registered address is: (single choice) [Show the card on page 5]

Township (town, street) where CAPI is loaded 1
Other townships (towns, streets) in the county (county-level city, district) where the CAPI is loaded 2
Other counties (county-level cities, districts) in the province where the CAPI is loaded .... 3 → skip to A4d
Other provinces .... 4 → skip to A4d
Account to be determined .... 5 → Jump question A5

A4c. In what year did you move your household registration here (district/county/county-level city where CAPI is loaded)?(Single choice)

Since the initial implementation of the current household registration system/birth .... 1 → skip question A5
Yes[_____|_____|_____|_____]Moved in 2

A4d. How long have you lived here (in this district/county/county-level city)?In what year?(Single choice) Less than half a year 1

More than half a year, yes[_____|_____|_____|........]For years 2

A5. Your religious belief is: (single choice) [show the card on page 6]

Christianity 1
Catholicism 2
Islamism 3
Taoism 4
Buddhism 5
Folk belief 6
Other religious beliefs (please specify) 7
No religious belief 8

A6a. Besides you, how many brothers and sisters with the same father and mother do you have?(including siblings who died after the age of 16)

Record: Brother has[_____|______]Number;Sisters have[____|______]Pieces

A6b.[Capi automatic screening only asks respondents with marriage experience, and those without marriage experience directly ask A6d] How many biological children do you have now?

Record: male[_____|______]Number;Female[___|______]Pieces

A6c.[Only ask respondents under 50 years old (born after 1969) who have children, and other respondents directly ask A6d] How many more do you want

A child?(Single choice) [Show the card on page 7]

One more 1
Two more 2
Three more 3
Maybe not 4
Definitely not 5
I haven't thought about it yet 6

A6d. [Everyone should answer] How many children do you think is the ideal number for a family?(The number of soft checks above 10 should be added to the capi)

Record:[_______|_____]Number;Among them, male[______|______________]Number;Female[___|______]Number; 99. It doesn't matter whether men or women

[CAPI: If you select 99 in the front, you can directly skip to B1]

Part B: Personal working conditions

Now I want to know about your production, work or business activities

B1. What is your current work situation? (single choice)

Working .... 1 → skip to B3a
Have a job, but currently take a vacation, study, or temporarily stop work or close down .... 2 → skip to B3a
jobless .... 3 → Capi loads the "work explanation" and asks B2a after confirming no work

The interviewer reads the following explanation of "work":

The work mentioned here refers to the last week: 1. I have been engaged in paid work for more than one hour;2. Working in his/her own family or family owned enterprises/institutions, although unpaid, but working more than 15 hours a week or more than 3 hours a day;3. Participate in agricultural production.If one of the above three conditions is met, it will be regarded as working.

Note: 1. Retirees, laid-off and unemployed persons, if they meet one of the above three

B2a. What is the main reason why you don't have a job at present?(Single choice) [Show the card on page 8]

Going to school .... 1 → skip to section B8
Incapacity .... 2 → skip to section B8
Retired 3
Not working after graduation 4
manage household chores 5
Loss of original job due to unit reasons (such as bankruptcy, restructuring, laid-off/early retirement/buyout length of service, dismissal, etc.) 6
Leaving the original job due to personal reasons (such as housework, health, resignation, etc.) 7
Contracted land is expropriated 8
Others (please specify) 9

B2b. How long have you been out of work

Record:[__________|_______]Year[_______|_______]Months
Capi: If the record is less than three years, ask: you have changed within three years_____Jobs (0 is allowed here)

B2c. Are you looking for a job to start your own business, or are you not looking for a job?(Single choice)

Looking for a job .... 1 → skip to B2e
Prepare to start your own business .... 2 → skip to B2e
Not only looking for a job, but also preparing to start their own business .... 3 → skip to B2e
Didn't look for a job and didn't plan to start their own business 4

B2d. Are you still going to work?(Single choice)

Intended to work .... 1 → skip to section B8
I'm not going to work .... 2 → skip to section B8

B2e. If you have a job now, can you go to work in two weeks?(Single choice)

can .... 1 → skip to section B8
No .... 2 → skip to section B8

[Note to interviewer: check B1, if "1-2" is selected, continue to ask B3a,

B3a. What is your current working status?(Single choice)

Currently only engaged in non-agricultural work 1
At present, they are mainly engaged in non-agricultural work, but also in agriculture 2
At present, it is mainly engaged in agriculture, but also engaged in non-agricultural work 3
At present, only farming .... 4 → Skip question B7

B3b. What is your main non-agricultural work (occupation) at present?(Please specify your position, position, type of work and work content. If your work activities belong to family operation, individual work alone or there is no specific work unit, please tell me what you have done.)

[Please refer to the Occupational Code Table for further inquiry and detailed records]

Record the name of the work unit (full name):______________________________ [||]

Record the specific job, title, administrative level, position and type of work:______________ (Capi: tree structure)

Record specific work content:______________________________

B3c. What industry does your non-agricultural work unit belong to?(For employees in the company, please state the specific name of the company/company, the type of production and business activities; for the security guards, labor workers, domestic service attendants, etc. sent by the labor dispatch agency, the labor dispatch agency is its unit; if there is no unit, the personal occupation is equal to the industry) [Please refer to the Industry Code Table for further inquiry and detailed records]

Record the name of the work unit (full name):______ CAPI directly loads the name of the work unit (full name) recorded in B3b____

[_____|_____]

Recording unit/company specific production and business activity type (industry):_____________

B3d. How many days have you worked in this non farm job on average every month since this year?[Please fill in the specific numbers on the horizontal line]

Record:[_______|_____]Days

B3e. How many hours have you worked in your non farm job on average every day since this year?[Please fill in the specific numbers on the horizontal line]

Record:[_______|_____]Hours

B3f. Since this year, how much income has your non farm work brought you per month?

[Please record the specific number, [N/A] is 9999997, [Reject Answer] is 9999999 (CAPI forms an option, and check N/A and N/A).If item d is year-end settlement, please calculate the average

project	Amount of money (yuan)						
	million	100000	ten thousand	thousand	hundred	ten	individual
a. Wages and salaries (including allowances and subsidies)	[___]	[___]	[___]	[___]	[___]	[___]	[___]
b. Bonus	[___]	[___]	[___]	[___]	[___]	[___]	[___]
c. Commission	[___]	[___]	[___]	[___]	[___]	[___]	[___]
d. Profits and dividends from business and investment	[___]	[___]	[___]	[___]	[___]	[___]	[___]
e. Other income (please specify)_________________	[___]	[___]	[___]	[___]	[___]	[___]	[___]

B3g1. Do you have a professional title or technical grade in your current non-agricultural work: (single choice) [show the card on page 9]

Professional title .... 1 → skip to B3g2
Technical grade .... 2 → skip to B3g3
No, but the professional title or technical level may be assessed in the future .... 3 → skip to B3g4
[not applicable] .... 9 → jump to B3g4
[Unclear] .... 10 → jump to B3g4
[No answer] .... 11 → skip to B3g4

B3g2. At present, in your non-agricultural work, your professional title is: (single choice) [show the card on page 10]

Senior professional title .... 1 → skip to B3g4
Intermediate professional title .... 2 → skip to B3g4
Primary professional title .... 3 → skip to B3g4

B3g3. At present, in your non-agricultural work, your technical level is: (single choice) [show the card on page 11]

Senior technician .... 1 → skip to B3g4
technician .... 2 → skip to B3g4
Senior engineer .... 3 → skip to B3g4
Intermediate worker .... 4 → skip to B3g4
Junior worker .... 5 → skip to B3g4

B3g4. Do you think the nature of your job is: (single choice) [show the card on page 12]

Work requiring high professional skills 1
Work requiring high professional skills 2
Work requiring some professional skills 3
Jobs without professional skills 4
Others (please specify) 5

Now please tell me some information about the unit/company where you are engaged in this non-agricultural work

[Note to interviewer: The organization should be an independent accounting organization with its own financial and personnel management functions. If the respondent's work organization is divided into many levels, and it is impossible to distinguish which level is his own organization, it can be prompted that the level of the respondent's salary relationship may be his

B4a. The unit/company where you are engaged in this non-agricultural work is: (single choice) [Show the card on page 13]

Party and government organs, people's organizations, and the military 01
State owned enterprises and state-owned holding enterprises 02
State owned/collective institutions 03
Collective enterprise 04
private enterprise 05
Foreign-funded enterprises 06
Individual businesses 07
Private institutions (private non enterprise units) 08
Community neighborhood committees, village committees and other autonomous organizations09
Others (please specify) 10
No unit 11
[Unclear]. 98

B4b. What is your status in the current non farm employment?(Single choice) [Show the card on page 14]

Employees or wage earners 1
Employer/boss (i.e. the owner/contributor/partner of the enterprise and employs others)
Capi continues to hire[_|_____|_____|_____]People.... 2 → Jump to B4e
Self employed workers (such as self-employed businesses and freelancers who do not employ others) 3 → Jump to B5
Domestic helper (working for his family/family's business, but not the boss).... 4 → Jump to B4d
Others (please specify) 5

B4c1. What kind of written contract do you currently sign with your work unit or employer?(Single choice) [Show the card on page 15]

Signed labor contract....1
Signed labor contract....2
Signed personnel contract.... 3 → Jump to B4d
Other contracts signed (please specify) .... 4 → Jump to B4d
No contract signed.... 5 → Jump to B4d
It is not necessary to sign labor/service contracts (such as civil servants or employees of state organs and public institutions) 6 → Jump to B4d
[Unclear]. . . . . 8 → Jump to B4d

B4c2. Which of the following written labor/service contracts do you currently sign?(Single choice) [Show the card on page 16]

Signed fixed term labor/labor contract....1
Signed open-ended labor/service contract....2
Signed labor/labor contract for probation period 3
Other labor/service contracts signed (please specify)....4
[Unclear]. 8

B4d. In the past 12 months, have you participated in the training provided by the unit to improve skills: (single choice)

Yes 1
No 2

B4e. In your current organization, your management activities are: (single choice) [Show the card on page 17]

Only manage others, not be managed by others (manage [|....| |__________]People) 1
Both manage others and be managed by others (manage [|....| |__________]People) 2
Only managed by others, not others 3

B4f. How long have you worked in your current company?

Record:[_______|______]Year[_____|______]Months

Capi: If the record is less than three years, ask: you have changed within three years___Jobs (0 is allowed here)

B5. How likely do you think you will lose your job in the next 6 months?(Single choice) [Show the card on page 18]

Quite possible 1
be on the cards 2
commonly 3
Unlikely 4
Absolutely impossible 5
[Unclear] 8

B6. Please use 1-10 points to express your satisfaction with the following aspects of the current job. 1 point means very dissatisfied, and 10 points means very satisfied: (single choice for each line)

	Extremely dissatisfied	Very satisfied	[Not applicable]
Overall job satisfaction	123456789	10	97

[Please note: B7 only asks respondents who answered "2-4" in B3a (that is, they have farming experience at present); respondents who do not have farming experience at present, please skip to

B7. What is your occupation in agriculture, forestry, animal husbandry and fishery in the last year?

[Please specify your work unit and business content, etc. If your work activities belong to farms, forest farms and other enterprise units, please tell me the name of your work unit; if your work activities belong to family business, individual work alone or there is no specific work unit, please

Record the name of the work unit (full name):________________

Record specific business content:________________ [||_]

[Please note: Part B8 asks all respondents]

Please note: B8a refers to the personal income of respondents in 2018.Filling requirements:

1. Ask visitors to record specific figures;"Not applicable", "Don't know/don't know" and "Refuse to answer" shall be checked accordingly;If there is a certain project, but there is no income on this item in 2018, this item will be recorded as 0.

2. After asking about the incomes, please compare the sum of the total incomes and the sub incomes. If the total incomes are less than the sum of the sub incomes, keep the original records. If

B8a. Please tell me your personal income last year (2018) is: [show the card on page 19]

project	Amount (yuan)							
	must	million	100000	ten thousand	thousand	hundred	ten	individual
a. Total revenue	[___]	[___]	[___]	[___]	[___]	[___]	[___]	[]
b. Wages, bonuses (including commissions, subsidies, etc.) and other labor remuneration income;Part time income Amateur labor income (such as writer's remuneration, class remuneration, various temporary helper's remuneration, etc.)	[___]	[___]	[___]	[___]	[___]	[___]	[___]	[]
c. Pension (paid by the company)	[___]	[___]	[___]	[___]	[___]	[___]	[___]	[]
d. Endowment insurance (from social insurance institutions)	[___]	[___]	[___]	[___]	[___]	[___]	[___]	[]
e. Social assistance income such as minimum living security fund and hardship allowance;Welfare provided by the village collective Income (such as dividends, subsidies, etc.)	[___]	[___]	[___]	[___]	[___]	[___]	[___]	[]
f. Net income from individual agricultural operation (including various agricultural subsidies)	[___]	[___]	[___]	[___]	[___]	[___]	[___]	[]
g. Profits and dividends from business and investment (employees holding shares of the enterprise should also fill in Answer)	[___]	[___]	[___]	[___]	[___]	[___]	[___]	[]
h. Income from gifts and inheritance of others	[___]	[___]	[___]	[___]	[___]	[___]	[___]	[]
i. Income from financial investment and wealth management (interest income from bonds, deposits, loans, etc., stock investment Income, dividends, bonus income, etc.)	[___]	[___]	[___]	[___]	[___]	[___]	[___]	[]
j. Other income (please specify)________	[___]	[___]	[___]	[___]	[___]	[___]	[___]	[]

[Note to interviewer: B8b asks all respondents]

[CAPI: For respondents who answered "2-4" in B3a (that is, they have agricultural experience at present), directly show B9, and automatically select 1 for B8b; for respondents who answered "1" in B3a (that is, they are only engaged in non-agricultural work), and respondents who are not currently employed in B1, show B8b]

B8b. Is there anyone in your family (including yourself) currently engaged in agriculture, forestry, animal husbandry, fishery and other labor?(Single choice)

Yes, someone (or himself) at home is engaged in agriculture, forestry, animal husbandry, fishery and other labor 1
No, but there is farmland/forest land/water surface in the family 2
No, there is no farmland/forest land/water surface at home .... 3 → skip to B10

B9. How many mu of farmland/forest land/water surface does your family have contracted?How many mu are actually operated at present?How many acres are idle?How many mu are transferred?How many mu are transferred out?[Please record the specific number, [Unclear] is 9998, [Refuse to Answer] is 9999, and check in the capi accordingly]

		a. Contracted area	b. Actual business area	c. Idle area	d. Transfer in area	e. Transfer out area
1	Cultivated land	[|	|] mu	[|	|] mu	[|	|] mu	[|	|] mu	[|	|] mu
2	woodland	[|	|] mu	[|	|] mu	[|	|] mu	[|	|] mu	[|	|] mu
3	surface of the water	[|	|] mu	[|	|] mu	[|	|] mu	[|	|] mu	[|	|] mu

Part C: Family economic situation

Next, we will ask you some questions about your family's production and living conditions, such as family residence, income, etc. If you are not very clear about these questions, you can also ask other family members who are familiar with the above conditions to answer them.

[Please note that if the interviewees do not understand the relevant questions in this section, they can ask other family members to answer on their behalf. If this question is answered by other family members, their answers should be recorded according to the interviewees. There is no upper limit on the number of

C1. How many self owned houses do you have at present?Record: There are [|] sets [If there is no self owned house, please fill in "0" set and ask C2; 99. Refuse to answer → ask C2]

Own housing	b. Building area (or homestead area): (m2)	c. Nature: [Show the card No. 23 Page]<br>1. Self built housing<br>2. Purchase commercial housing<br>3. Purchase all kinds of policy support housing<br>4. Purchase the original public house<br>5. Purchase of houses with small property rights<br>6. Purchase of rural private housing<br>7. other<br>8. [Unclear]	d. Type of region: [Show the card on page 24]<br>1. Urban area of municipality directly under the Central Government<br>2. Urban area of provincial capital<br>3. Prefecture/county-level city<br>4. county town<br>5. Towns outside the city/county seat<br>6. countryside	e. Time of purchase (or self construction): (year)<br>9998. [Unclear]	f. Price at the time of purchase (or self construction): (10000 yuan) [Please estimate, if less than 10000 yuan, fill in as 10000 yuan]<br>9998. [Unclear]	g. Present value of house: (10000 yuan) [Please estimate, if less than 10000 yuan, fill in as 10000 yuan]<br>9998. [Unclear]	h. The house at the time of investigation: [show the card on page 25]<br>1. I live<br>2. If you do not live in person, your family or other relatives will live<br>3. Renting others<br>4. Lending to others<br>5. Nobody lives<br>6. Pending house
First set	[|||]	[___]	[___]	[|	|] years	[|	|] 10000 yuan	[|	|] 10000 yuan	[___]
Second set	[|||]	[___]	[___]	[|	|] years	[| _	|] 10000 yuan	[|	|] 10000 yuan	[___]
Third set	[|||]	[___]	[___]	[|	|] years	[|	|] 10000 yuan	[|	|] 10000 yuan	[___]
The fourth set	[|||]	[___]	[___]	[|	|] years	[|	|] 10000 yuan	[|	|] 10000 yuan	[___]
…	…	…	…	…	…	…	

C2. The suite you currently live in is: [Show the card on page 26]

Own housing 1

Capi: If you choose this option, you need to ask which house you currently live in. capi

If there is only one set, cappi automatically fills in 1

Low rent housing/public rental housing 2

Family room 3

Private room 4

Collective dormitory 5

Others (please specify) 6

[Unclear] 8

C3a. Last year (2018), your family's overall income and expenditure were: (single choice)

Income exceeds expenditure 1

Break even 2

Less income than expenditure 3

[Hard to say] 8

C3b. Please tell me about your family's living consumption expenditure last year (2018): [show the card on page 27]

project	Amount (yuan)						
	million	100000	ten thousand	thousand	hundred	ten	individual
a. Total living expenditure	[__]	[__]	[__]	[__]	[__]	[__]	[__]
b. Food expenditure (including food expenditure for going out; estimate and calculate the price of self-produced food)	[__]	[__]	[__]	[__]	[__]	[__]	[__]
c. Clothing expenses (clothes, shoes and hats, etc.)	[__]	[__]	[__]	[__]	[__]	[__]	[__]
d. Payment of rent	[__]	[__]	[__]	[__]	[__]	[__]	[__]
e. Down payment for house purchase and amortization of house loans (excluding down payment in 2018)	[__]	[__]	[__]	[__]	[__]	[__]	[__]
f. Electricity, water, gas (coal), property, heating	[__]	[__]	[__]	[__]	[__]	[__]	[__]
g. Purchase expenses of household appliances, furniture, household vehicles, etc	[__]	[__]	[__]	[__]	[__]	[__]	[__]
h. Medical care expenses (such as medical treatment, hospitalization, drug purchase, etc.)	[__]	[__]	[__]	[__]	[__]	[__]	[__]
i. Communication expenses (such as fixed line telephone/mobile phone/PHS phone charges, computer internet charges, etc.)	[__]	[__]	[__]	[__]	[__]	[__]	[__]
j. Transportation expenses (such as transportation fees for off duty, gasoline fees for household vehicles, maintenance fees, road maintenance fees, road and bridge fees, etc.)	[__]	[__]	[__]	[__]	[__]	[__]	[__]
k. Education expenditure (such as tuition, miscellaneous fees, stationery fees, after-school tutoring fees, school accommodation fees, etc., but school food expenditure is included in family food expenditure)	[__]	[__]	[__]	[__]	[__]	[__]	[__]
l. Expenditure on culture, entertainment and tourism	[__]	[__]	[__]	[__]	[__]	[__]	[__]
m. Domestic service expenditure (Note: refers to the household expenditure of respondents)							
n. Expenditure for supporting or caring for relatives who do not live together (such as parents and other elderly people)	[__]	[__]	[__]	[__]	[__]	[__]	[__]
o. Expenditure on weddings and funerals;Personal relationship expenditure (such as gifts, cash, etc.)	[__]	[__]	[__]	[__]	[__]	[__]	[__]
p. Other expenses (please specify)______________	[__]	[__]	[__]	[__]	[__]	[__]	[__]

C3c. Please record that the expenditure recorded in C3b is from the family table

______________________________________Expenditures for

[Note to Capi: check the family members in Table A1a]

[Please note: C4a refers to the family income of respondents in 2018. Filling requirements:

1. Ask visitors to record specific figures;Check the following options: "Not Applicable" is recorded as 99999997;"Don't know/don't know" is recorded as 99999998;The record of "Reject Answer" is 99999999;If there is a certain project, but there is no income on this item in 2018, this item will be recorded as 0.

2. After asking about the income of each item, the investigator should compare the sum of the total income and the income of each item. If the total income is less than the sum of the income of

C4a. Please tell me the income of your family last year (2018): [show the card on page 28]

project	Amount (yuan)							
	must	million	100000	ten thousand	thousand	hundred	ten	individual
a. Total income of your family	[__]	[__]	[__]	[__]	[__]	[__]	[__]	[__]
b. Your family's salary income (including salary, bonus, allowance, holiday benefits, etc., if any , Please calculate the discount;Note that retirement pension is not included)	[__]	[__]	[__]	[__]	[__]	[__]	[__]	[__]
c. Net income from agricultural operation	[__]	[__]	[__]	[__]	[__]	[__]	[__]	[__]
d. Income from business and factory operation	[__]	[__]	[__]	[__]	[__]	[__]	[__]	[__]
e. Income from selling/renting houses and land	[__]	[__]	[__]	[__]	[__]	[__]	[__]	[__]
f. Income from household financial investment and wealth management (interest income from bonds, deposits, loans, etc., stock investment Capital income, dividends, bonus income, etc.)	[__]	[__]	[__]	[__]	[__]	[__]	[__]	[__]
g. Family member pension, endowment insurance, unemployment insurance, work-related injury insurance, maternity insurance Social insurance income such as insurance premium	[__]	[__]	[__]	[__]	[__]	[__]	[__]	[__]
h. Income from reimbursement of medical expenses of family members	[__]	[__]	[__]	[__]	[__]	[__]	[__]	[__]
i. Income from social assistance provided by the government, work units and other social institutions (such as minimum living security, subsidies for difficulties, disease relief, disaster relief, school scholarships/grants, poverty Student assistance, etc.)	[__]	[__]	[__]	[__]	[__]	[__]	[__]	[__]
j. Production and operation subsidies and policy support income provided by the government (such as agricultural subsidies and tax relief) Etc.)	[__]	[__]	[__]	[__]	[__]	[__]	[__]	[__]
k. Welfare income provided by neighborhood committees and village committees (such as collective production and operation dividends, non relief Subsidies, etc.)	[__]	[__]	[__]	[__]	[__]	[__]	[__]	[__]
l. Other income (please specify)______________________________	[__]	[__]	[__]	[__]	[__]	[__]	[__]	[__]

C4b. Please record that the income recorded in C4a is from the family table__Income of

[Note to Capi: check the family members in Table A1a]

C5 Parents and Spouses (CAPI Random Volume A)

C5-1. We have already learned about your parents and your spouse.Now I want to know more about their occupation and work.

A. Relationship with respondents:	B. Account type	C. What is your current or last occupation	D. What type of organization is your current or final job?[Show the card on page 29]	E. Identity in current or final work [show the card on page 30]	F. Professional title and technical qualification of current or final work [show the card on page 31]	G. Management position in current or final work unit
	1. Non-agricultural<br>2. Agriculture<br>3. Others (please specify____)<br><br>Unified numbering<br><br>***[Unclear]***	1. Non-agricultural<br>2. Agriculture<br>3. Others (please specify _______)<br><br>Unified numbering<br><br>***[Unclear]***	1. Party and government organs, people's organizations, and the military<br>2. State owned enterprises and state-owned holding enterprises<br>3. State owned/collective institutions<br>4. Collective enterprise<br>5. private enterprise<br>6. Foreign-funded enterprises<br>7. Individual businesses<br>8. Private institutions (private non enterprise units)<br>9. Community neighborhood committees, village committees and other autonomous organizations<br>10. Non farm self-employed workers, no unit<br>11. Engaged in agricultural labor without unit<br>12. Others (please specify)________<br>Unified numbering[Not applicable] Unified numbering[Unclear]<br>Unified numbering[No answer]	1. a farmer<br>2. Employees or wage earners<br>3. Employer/boss (i.e. the owner/investor/partner of the enterprise and employs others)<br>4. Self employed workers (self-employed businesses and freelancers who do not employ others in non-agricultural employment)<br>Business owner)<br>5. Family helper (working for his family/family's enterprise in non-agricultural employment, but not the boss)<br>6. Other (please specify)<br>unified numbers[Not applicable] Unified numbering[Unclear]<br>Unified numbering[No answer]<br>_________	1. Senior professional title<br>2. Intermediate professional title<br>3. Primary professional title<br>4. Senior technician<br>5. technician<br>6. Senior engineer<br>7. Intermediate worker<br>8. Junior worker<br>9. No professional title and technical level<br><br>Unified numbering<br>***[Not applicable]***<br>***[Unclear]***<br>***[No answer]***	1. Senior managers<br>2. middle managers<br>3. Lower management<br>4. Ordinary staff<br><br>Unified numbering<br>***[Not applicable]***<br>***[Unclear]***<br>***[No answer]***
father						
mother						
spouse						

[Note to Capi: The last line "spouse" will be loaded only when A1a respondents choose 2, 3 and 5 for their marital status]

C5-2: Please answer the information about your parents at the following times

A. Relationship with respondents:	B. Current account type	C. What was your occupation at that time	D. What kind of organization was the work at that time?[Show the card on page 29]	E. Identity in the work at that time [Show the card on page 30]	F. Professional title of current or final work [show card on page 31] and technical qualification [show card on page 74]	G. Management position in the work unit at that time
	1. Non-agricultural<br>2. Agriculture<br>3. Others (please specify)<br><br>Unified numbering<br>***[Unclear]***	1. Non-agricultural<br>2. Agriculture<br>3. Others (please specify_____)<br><br>Unified numbering<br>***[Unclear]***	1. Party and government organs, people's organizations, and the military<br>2. State owned enterprises and state-owned holding enterprises<br>3. State owned/collective institutions<br>4. Collective enterprise<br>5. private enterprise<br>6. Three capital enterprises<br>7. Individual businesses<br>8. Private institutions (private non enterprises Business unit)<br>9. Community neighborhood committees, village committees and other autonomous organizations<br>10. Non farm self-employed workers, no single position<br>11. Engaged in agricultural labor without unit<br>12. Others (please specify)_________<br><br>Unified numbering[Not applicable]<br>Unified numbering[Unclear]<br>Unified numbering[No answer]	1. a farmer<br>2. Employees or wage earners<br>3. Employer/boss (i.e. the owner/contributor/partner of the enterprise and employs others)<br>4. Self employed workers (self-employed businesses and freelancers who do not employ others in non-agricultural employment)<br>5. Family helper (working for his family/family's enterprise in non-agricultural employment, but not the boss)<br>6. Others (please specify)_________<br>Unified numbering[Not applicable] Unified numbering[Unclear] Unified numbering[No answer]	1. Senior professional title<br>2. Intermediate professional title<br>3. Primary professional title<br>4. Senior technician<br>5. technician<br>6. Senior engineer<br><br>7. Intermediate worker<br>8. Junior worker<br>9. No professional title and technical level<br><br>Unified numbering[Not applicable] Unified numbering[Unclear]<br>Unified numbering[No answer]	1. Senior managers<br>2. middle managers<br>3. Lower management<br>4. Ordinary staff<br><br>Unified numbering[Not applicable] Unified numbering[Unclear] Unified numbering[No answer]
Your father's situation when you were 14 years old						
Your mother's situation when you were 14 years old						
What happened to your father when you started working						
What happened to your mother when you started working						

Part D: Living Conditions

Next, we would like to know if you have any problems in your life and your evaluation of life.
D1. Which of the following life problems have you or your family encountered in the past 12 months?(Multiple choices are allowed) [Show the card on page 32]

Poor housing conditions, unable to build/buy houses 01
Children's education costs are too high to bear 02
Inconsistent family relationship (such as divorce, separation, bad relationship between mother-in-law and daughter-in-law, etc.) 03
The medical expenditure is too large to bear 04
Rising prices will affect living standards 05
Low family income and difficulties in daily life 06
Unemployed, unemployed or unstable family members 07
The burden of supporting the elderly is too heavy 08
The expenditure on family friendship is too large to bear 09
Encounter cheating, theft, robbery and other criminal events 10
Serious environmental pollution in residential areas 11
Other life pressures and difficulties (please specify______) 12
No such life problems 13

D2a. Please use 1-10 points to express your satisfaction with the following items: 1 point means very dissatisfied, 10 points means very satisfied: (single choice for each line) [show the card on page 33]

		**Extremely dissatisfied Very satisfied**
1	Your family relationship	123456789 10
2	Your family's financial situation	123456789 10
3	Your education level	123456789 10
4	Your leisure/entertainment/cultural activities	123456789 10
5	Your social life	123456789 10
6	Overall, your satisfaction with life	123456789 10

(CAPI random volume B) D2b. Do you agree with the following statement.(Single choice for each line) [Show the card on page 34] CAPI provides random control of jumping questions

		**very agree!**	**compare agree!**	**Not very agree!**	**No agree!**	**[No Good to say]**
1	My life roughly conforms to my ideal.	1	2	3	4	8
2	Even if life can start all over again, I don't want to change anything.	1	2	3	4	8
3	In general, I will achieve financial freedom in my life and allow me to enjoy my life.	1	2	3	4	8
4	On the whole, I am a happy person.	1	2	3	4	8

(CAPI random volume B) D2cIn the past six months, do the following statements fit your situation?(Single choice for each line) [Show the card on page 35]

(CAPI provides random control of jumping questions, only asking questions B3a. Your current work status is to choose 1-2 respondents, and the others will jump to D3a directly)

		accord with	Non conformance
1	I am always tired when I come home after work, and I have no energy to do housework (take care of children)	1	2
2	I spend most of my time at work, and it's hard for me to take on family responsibilities	1	2
3	Doing housework (taking care of children) is very tiring. I always can't finish my work well	1	2
4	It's hard for me to concentrate on my work because I have to do my family duty	1	2

D3a. What level do you think your socio-economic status generally belongs to in the local area?(Single choice) [Show the card on page 36]

Upper 1
so-so 2
Medium 3
lower-middle 4
Next 5
[Hard to say] 8

D3b. What level do you think your socio-economic status was at 5 years ago?(Single choice) [Show the card on page 36]

Upper 1
so-so 2
Medium 3
lower-middle 4
Next 5
[Hard to say] 8

D3c. What level do you think your socio-economic status will belong to in the next five years?(Single choice) [Show the card on page 36]

Upper 1
so-so 2
Medium 3
lower-middle 4
Next 5
[Hard to say] 8

D4a. Now the Internet is more popular. People can use mobile phones and computers to access the Internet. Do you usually use computers or mobile phones to watch news, use WeChat and other activities?(Single choice)

Upper 1
No .... 2 → skip to D4b3

D4b1. How often do you do the following activities online: (single choice for each line) [Show the card on page 37]

		Almost every day	More than once a week	At least one week once	At least one month once	Several times a year	never
1	Browse current political information (such as party and government news)	1	2	3	4	5	0
2	Entertainment and leisure (such as playing online games/listening to music/watching videos/reading novels)	1	2	3	4	5	0
3	Chat and make friends (such as WeChat and other dating activities)	1	2	3	4	5	0
4	Business or work	1	2	3	4	5	0
5	Learning education	1	2	3	4	5	0
6	Online shopping/life services (such as online shopping, takeout, map navigation<br>Figure Positioning, etc.)	1	2	3	4	5	0
7	Investment and Financing	1	2	3	4	5	0
8	Others (please specify)	1	2	3	4	5	0

D4b2. Which of the following online social groups/circles have you joined in the past two years?[Show the card on page 38]

Social circle between relatives 01
Social circle between friends 02
Social circle between neighbors (e.g. owners) 03
Colleagues 04
Religious belief group 05
Fellow townsmen 06
Students and alumni 07
Interest groups (such as sports and entertainment groups, shopping groups, etc.) 08
Public welfare associations (such as family committees, volunteers, environmental protection organizations, etc.) 09
Industry group, peer group, association group (such as chambers of commerce, rural cooperative organizations, professional associations, industry associations, etc.)ten
Rights group 11
Other groups (please specify___) 12
None of the above 13

D4b3. Which of the following groups have you participated in offline activities in the past two years?(Multiple choices are allowed) [Show the card on page 39]

religious community 1
Clan Association 2
The Alumni Association 3
Recreational and sports interest organizations 4
Non governmental public welfare organizations (such as volunteers, owners' committees, environmental protection organizations) 5
Professional organizations (such as chambers of commerce, rural cooperative organizations, professional associations, industry associations, etc.) 6
Other groups (please specify) 7
None of the above 8

D4c. Do you agree with the following statements?(Single choice for each line) [Show the card on page 40]

		I agree	Comparatively agree	Disagree	Disagree	[Not good Say]
1	Compared with TV, radio and newspapers, the information on the Internet is more comprehensive and in-depth	1	2	3	4	8
2	News on the Internet is less authentic than that on TV, radio and newspapers	1	2	3	4	8
3	At present, the Internet is the best channel to express public opinion and reflect the real situation of society	1	2	3	4	8
4	The Internet can indeed play a certain role in supervising the work of the government	1	2	3	4	8

CAPI provides jump question random control:

(CAPI random volume B) D5aIn the area where you currently live (residential area/county/county-level city loaded by capi), are the following phenomena serious?(Single choice for each line) [Show the card on page 41]

		Very serious	Relatively serious	Not too serious	No such phenomenon	[Hard to say]
1	air pollution	1	2	3	4	8
2	water pollution	1	2	3	4	8
3	noise pollution	1	2	3	4	8
4	Other environmental pollution (such as land pollution, electromagnetic<br>Radiation pollution, etc.) (please specify)________	1	2	3	4	8

(CAPI random volume B) D5bPlease use a scale of 1-10 to express your satisfaction with the environmental conditions of your current residence (residential area/county/county-level city loaded by capi). 1 point means very dissatisfied, 10 points means very satisfied: (single choice)

	Extremely dissatisfied	Very satisfied
Environmental conditions of residence	123456789	10

(CAPI random volume B) D5c. Is the following statement consistent with your daily situation or ideas?Is it completely consistent, relatively consistent, not very consistent, or completely inconsistent?(Single choice for each line) [Show the card on page 42]

		Fully compliant	More consistent	Not very consistent	Completely inconformity	[Hard to say]
1	For China, economic development is more important than environmental protection	1	2	3	4	8
2	It's the government's responsibility to protect the environment, which has nothing to do with me	1	2	3	4	8
3	If I have time, I am very willing to participate in environmental protection organizations	1	2	3	4	8
4	I don't understand environmental issues, and I don't have the ability to comment	1	2	3	4	8

(CAPI random volume B) D5dIf the government department plans to build a garbage disposal station near your community, does the following statement match your idea?Is it completely consistent, relatively consistent, not very consistent, or completely inconsistent?(Single choice for each line) [Show the card on page 43]

		Fully compliant	More consistent	Not very consistent	Completely inconformity	[Not good Say]
1	I will definitely stand up against the construction of the garbage station	1	2	3	4	8
2	I don't believe what the government said, "The garbage station has little pollution and no impact on residents"	1	2	3	4	8
3	I don't believe the words of environmental protection experts that "the garbage station has little pollution and no impact on residents"	1	2	3	4	8

Part E: Social Security

Next, we would like to know about your social security situation and your evaluation of social security.

E1a. Do you currently participate in the following social security?(Single choice) [Show the card on page 44]

E1b.[If there is endowment insurance or medical insurance] Which of the following social security do you participate in?[Show the card on page 45]

	E1a. Do you currently have the following social security? (Single choice)			E1b. Which of the following social security do you have?(multiple choice)
	yes	No,	[Unclear]	
A. Pension insurance or pension	1	2	8	1. Basic endowment insurance for urban employees<br>2. Social endowment insurance for urban and rural residents<br>3. Pension<br>4. Rural social endowment insurance (i.e. new rural insurance)
B. Medical insurance or free medical treatment	1	2	8	1. Basic medical insurance for urban employees<br>2. Basic medical insurance for urban residents<br>3. Free medical treatment<br>4. New rural cooperative medical insurance (new rural cooperative medical insurance)<br>5. Serious disease insurance for urban and rural residents
C. Unemployment insurance	1	2	8	
D. Industrial injury insurance	1	2	8	
E. Maternity insurance	1	2	8	
F. Urban and rural minimum living security (i.e Subsistence allowance)	1	2	8	

E1c. Please use 1-10 points to express your evaluation of the following social security conditions provided by the government to the people. 1 point means very dissatisfied, 10 points means very satisfied: (single choice for each line) [show the card on page 46]

		Extremely dissatisfied									Very satisfied	[Unclear]
1	Pension security	1	2	3	4	5	6	7	8	9	10	98
2	medical security	1	2	3	4	5	6	7	8	9	10	98
3	Employment security	1	2	3	4	5	6	7	8	9	10	98
4	Urban and rural minimum living security	1	2	3	4	5	6	7	8	9	10	98
5	Government provided affordable housing, public rental housing, low rent housing and other basic housing security	1	2	3	4	5	6	7	8	9	10	98
6	Overall social security	1	2	3	4	5	6	7	8	9	10	98

E2. Do you agree with the following opinions: (single choice for each line) [show the card on page 47]

	viewpoint	very disagree	Not very agree!	compare agree!	very agree!	[Hard to say]
1	Social security is the basic responsibility of the government and should not be borne by ordinary people	1	2	3	4	8
2	The current level of social security is too low to play the role of security	1	2	3	4	8
3	I am dissatisfied with the social security provided by the government	1	2	3	4	8

Part F: Social trust and social equity

Next, we would like to know your views on some current social problems.

F1a. Excuse me, do you trust the following institutions?(Single choice for each line) [Show the card on page 48] (note to capi: only option 5 is not applicable)

		Total distrust	Not trusted	More trust	Very trusted	[Hard to say]	[Not applicable]
1	central government	1	2	3	4	8	
2	District and county governments	1	2	3	4	8	
3	Township government	1	2	3	4	8	
4	Workers, youth, women and other mass organizations	1	2	3	4	8	
5	Work unit/company	1	2	3	4	8	9
6	mission	1	2	3	4	8	
7	news media	1	2	3	4	8	
8	bank	1	2	3	4	8	
9	insurance company	1	2	3	4	8	
10	hospital	1	2	3	4	8	
11	court	1	2	3	4	8	
12	police	1	2	3	4	8	

F1b. Please use 1-10 points to express your evaluation of the trust level between people. 1 point means very distrust, and 10 points means very trust: (single choice)

	Very distrustful	Very trusted
Current level of trust between people	123456789	10

F1c. To what extent do you agree with the following statements?(Single choice for each line) [Show the card on page 49]

		I agree	Comparatively agree	Disagree	Disagree	[Hard to say]
1	I'm worried that personal information will be collected and used casually by businesses	1	2	3	4	5
2	Most businesses can reasonably use consumer information	1	2	3	4	5
3	Current laws and regulations have done more to protect consumers' privacy than<br>Good regulations	1	2	3	4	5

F2. Do you think the unfair treatment in the following aspects of society is serious now: (single choice for each line) [show the card on page 50]

		Very serious	Relatively serious	Not too serious	No such problem	[Hard to say]
1	Age	1	2	3	4	8
2	Gender	1	2	3	4	8
3	Race/ethnicity	1	2	3	4	8
4	registered residence	1	2	3	4	8
5	Religion	1	2	3	4	8
6	Education	1	2	3	4	8
7	occupation	1	2	3	4	8
8	Family background and social relations	1	2	3	4	8

F3a. In terms of your personal beliefs, can you accept the following groups: (single choice per line) [show the card on page 51]

		Very unacceptable	Not very receptive	More acceptable	Very receptive	[Hard to say]
1	Premarital cohabitant	1	2	3	4	8
2	homosexuality	1	2	3	4	8
3	Beggar	1	2	3	4	8
4	Releaser after serving a sentence	1	2	3	4	8
5	People with different religious beliefs	1	2	3	4	8
6	AIDS patients	1	2	3	4	8

F3b. Please use 1-10 points to express your evaluation of the tolerance of the current society. 1 point means very intolerant, 10 points means very tolerant: (single choice)

	Very intolerant	Very tolerant
**The tolerance of the present society**	123456789	10

F4a. Do you agree with the following statement.(Single choice for each line) [Show the card on page 52]

		very agree!	compare agree!	Not very agree!	No agree!	[No Good to say]
1	The social environment in which I was born has a great impact on my future	1	2	3	4	8
2	In today's society, the possibility of "starting a business from scratch" is still great	1	2	3	4	8
3	In a just society, income should be equally distributed among the members of society	1	2	3	4	8
4	When being treated unfairly by the society, it is better to tolerate	1	2	3	4	8
5	In China, only those who have power, money and connections can live a good life	1	2	3	4	8

F4b1. How fair do you think the following aspects of current social life are?(Single choice for each line) [Show the card on page 53]

		Very unfair	Not fair	Fairer	Very fair	[Hard to say]
1	College Entrance Examination System	1	2	3	4	8
2	Political rights actually enjoyed by citizens	1	2	3	4	8
3	Justice and law enforcement	1	2	3	4	8
4	Public health	1	2	3	4	8
5	Jobs and employment opportunities	1	2	3	4	8
6	Wealth and income distribution	1	2	3	4	8
7	Social security benefits such as pension	1	2	3	4	8
8	Rights and treatment between urban and rural areas	1	2	3	4	8

F4b2. In any society, there are a certain proportion of poor people. What do you think is the main reason why the poor will fall into poverty?(Select 3 at most
Items, and sort them) [Show the card on page 54]

		The main reason	Second main reason	Third main reason
1	Lack of ability and talent	1	1	1
2	Bad luck	2	2	2
3	Poor family conditions	3	3	3
4	lazy	4	4	4
5	Poor health and disability	5	5	5
6	Low education	6	6	6
7	Laws and policies lack equal protection for the poor	7	7	7

8	Lack of social relations	8	8	8

F4b3. In any society, there are a certain proportion of rich people. What do you think is the main reason why rich people can obtain wealth?(Select 3 at most Items and sort them) [Show the card on page 55]

		The main reason	Second main reason	Third main reason
1	Ability and ability	1	1	1
2	in luck	2	2	2
3	Good family background	3	3	3
4	Work hard	4	4	4
5	Have social relations	5	5	5
6	High education	6	6	6
7	Laws and policies favor the rich	7	7	7
8	taking risk	8	8	8
9	Violate laws and disciplines, and go astray	9	9	9

F4b4. Please use a scale of 1-10 to express your assessment of the overall fairness and justice of the current society. 1 point means very unfair, and 10 points means very fair: (single choice)

	Very unfair	Very fair
On the whole, you are fair to the current society	123456789	10

(CAPI random volume B)

F4b5?(Select 3 items at most) [Show the card on page 56]

- Now most laws are unfair and unjust 01
- Now people in the society only think about themselves........ 02
- Most of the court decisions are unfair........ 03
- Most people in society do not have high-quality medical security 04
- Only a small number of people benefited from the reform 05
- The current social injustice is mainly caused by the corruption of party and government cadres 06
- The government has not provided sufficient social security for vulnerable groups 07
- There are quite a few people in our society who have no chance to receive good education 08
- Now society is unfair and unjust in the distribution of material wealth........ 09
- No society is completely fair and just 10
- Other reasons (please specify________） 11
- It's hard to say 12

(CAPI random

F4c. What do you think is the most important of the following options for the current Chinese society?[Show the card on page 57] (provided by CAPI

Jump question

- Maintain social order 1
- Give the general public more voice in important government decisions........ 2
- Combating price rise........ 3
- Protection of free speech 4
- Others (please specify_____） 5
- be unable to explain clearly 6

Part G: Social Values and Social Evaluation

Now we would like to know your comments on the current social climate, local government work and other issues.

G1. To what extent do you agree with the following statements?(Single choice for each line) [Show the card on page 58]

		I agree	Comparatively agree	Disagree	Disagree	[Hard to say]
1	Now most people have no faith	1	2	3	4	8
2	I am always proud of the achievements of my country	1	2	3	4	8
3	If there is a next life, I would still like to be a Chinese	1	2	3	4	8
4	Every Chinese has the same opportunity to get wealth and happiness	1	2	3	4	8
5	Without the Communist Party, China would fall into chaos	1	2	3	4	8

G2. Please use 1-10 points to express your evaluation of the following items. 1 point means very bad, 10 points means very good: (single choice for each line)

		Very bad	very nice
1	The general moral level of people in the society	123456789	10
2	People's compliance level in the society	123456789	10

G3. Do you think (CAPI automatically loads the first level name of the PSU where the respondents live) the government has done a good job in the following aspects?(Single choice for each line) [Show the card on page 59]

		very good	Quite good	just so so	Very bad	[Unclear]
1	Provide medical and health services	1	2	3	4	8
2	Provide social security for the masses	1	2	3	4	8
3	Protect the environment and control pollution	1	2	3	4	8
4	Safeguarding citizens' political rights	1	2	3	4	8
5	Fight against crime and maintain public order	1	2	3	4	8
6	Clean and honest, punish corruption	1	2	3	4	8
7	Act in accordance with the law and enforce the law fairly	1	2	3	4	8
8	Develop economy and increase people's income	1	2	3	4	8
9	Expand employment and increase employment opportunities	1	2	3	4	8
10	Open government information and improve the transparency of government work	1	2	3	4	8
11	Have a sense of service and can respond to people's demands in a timely manner	1	2	3	4	8
12	Provide high-quality education resources and ensure education fairness	1	2	3	4	8
13	Ensure food and drug safety	1	2	3	4	8
14	In general, the work of local governments	1	2	3	4	8

Now please listen to a few scenarios, and then select the options that are more suitable for your personal situation

[Please read out the following scenarios one by one, clearly explain the views of A and B, and then ask the interviewees to choose which view they agree with more or

Practice]

G4a. A local government needs to carry out land acquisition and demolition due to water conservancy construction. Party A believes that only when the land acquisition and demolition funds provided by the government fully meet their own requirements will they move away, otherwise they will not move away;B believes that since the government wants to land for water conservancy construction, as ordinary people, it should obey the government's arrangements.Which of the ideas of A and B mentioned above is closer to your idea?

I quite agree with A's statement..1

I quite agree with B's statement..2

G4b. Party A and Party B plan to jointly set up a factory, but they are not qualified to operate and cannot obtain a business license.A believes that if you want to get a business license, you should find some connections and give some benefits to the staff;Party B believes that it does not comply with the relevant provisions on factory operation, and will consider it after the conditions are met.If it is you, which of the ideas of A and B mentioned above is closer to your idea?

I quite agree with A's statement..1

I quite agree with B's statement 2

G5. What do you think is the most important social problem in China?(Select 3 items at most) [Show the card on page 60]

	problem	choice
1	Employment and unemployment	01
2	Difficult and expensive to see a doctor	02
3	Old age security	03
4	Educational charges	04
5	The problem of the large gap between rich and poor	05
6	Price rise	06
7	Housing price is too high	07
8	Social security issues	08
9	Declining social trust	09
10	Corruption	10
11	Environmental pollution	11
12	Food and Drug Safety	12
13	Unfair compensation for land acquisition and demolition	13
14	Unfair treatment of migrant workers in cities	14
15	Others (please specify)___________	15

G6. Please use a scale of 1-10 to express your assessment of the overall situation of the current society. 1 point means very bad, 10 points means very good: (single choice)

	Very bad	very nice
In general, your assessment of the current society	123456789	10

G7. Which of the following characteristics do you think a good society should include?(Please select 5 items at most) [Show the card on page 61]

Respect the Constitution 01
Be rich and strong........02
democratic........03
innovate 04
civilization 05
harmonious 06
Inclusion........07
free 08
equality 09
Advocate science 10
fair 11
rule by law 12
Respect for human rights........13
Patriotic........14
be dedicated 15
Collectivism 16
sincerity........17
friendly........18
unite 19
Others (please specify) 20
be unable to explain clearly 21

Part H: Social and Political Participation

Now we would like to know about your participation in social activities.

H1a. Have you participated in the following events in the past two years?(Multiple choices are allowed) [Show the card on page 62]
(0 for H1a in capi, H1b for continuation, 1 for H1a, H1c for continuation)

H1b. If you have not participated, are you willing to participate?(Single choice per line)

		H1a. Have you participated in the following events in the past two years? (Multiple choices are allowed) 1. Attended 0. No participation		H1b. If you have not participated, are you willing to participate? (Single choice)		
				Willing to participate	Not willing to participate	[Hard to say]
1	Reflect social problems to newspapers, radio stations, online forums and other media	1	0 --->	1	2	8
2	Reflect opinions to government departments	1	0 --->	1	2	8
3	Participate in the election of village (neighborhood) committee	1	0 --->	1	2	8
4	Participate in major decision-making discussions of the village/unit	1	0 --->	1	2	8
5	Participate in online/offline collective rights protection actions	1	0 --->	1	2	8

H1c_a. In the last five years, have you participated in the "Vote to elect deputies to district and county people's congresses"

Participated .... 1 → skip to H2

Didn't participate 2

H1c_b. If you have not participated, are you willing to participate in the next election of deputies to district and county people's congresses?(Single choice)

Willing to participate 1

Not willing to participate 2

H2. Do you agree with the following statement: (single choice for each line) [show the card on page 63]

		I agree	Comparatively agree	Disagree	Disagree	[Hard to say]
1	I have the ability and knowledge to comment on politics	1	2	3	4	8
2	My freedom of speech will be restricted by the government	1	2	3	4	8
3	It's no use for the people to participate in political activities, and they can't have any fundamental impact on the government	1	2	3	4	8
4	The common people should obey the government, and the lower level should obey the higher level	1	2	3	4	8
5	The government is in charge of national affairs, so people don't need to think too much	1	2	3	4	8

Part I: Volunteering

Let's learn something about your volunteer service

I1. Generally speaking, what we mean by voluntary service is labor services provided voluntarily and free of charge for people or things other than family members, not donations such as money, goods and blood.

Which of the following volunteer services have you participated in in the past year?(Multiple choices are allowed) [Show the card on page 64]

Care for children........01
Youth counselling........02
Elderly care........03
Women's rights protection........04
Help the disabled........05
Teaching assistant........06
Poverty alleviation........07
medical care........08
legal aid........09
environmental protection........10
Rescue and disaster relief........11
international aid........12
Others (please specify supporting the army and giving preferential treatment to families, educational aid, psychological consultation, large-scale social activities, etc.)........13
None of the above........14 → (skip to I5)

I2. Who organized the volunteer services you have participated in in the past year?(Multiple choices are allowed) [Show the card on page 65]

Initiated by individuals........1
Workers, young women and other mass organizations........2
Work unit/school/organization/institution........3
Community (Village) Committee........4
Government departments and relevant institutions........5
volunteer service organization........6
Others (please specify)........7

I3. You have participated in______________Volunteer service activities, about_hour

I4. Have you ever provided volunteer service activities through the Internet?

Yes 1
No 2

I5. Do you need to have some professional knowledge or skills to participate in voluntary services?

All required........1
Most need........2
Individual needs........3
Not at all........4

I6. Have you participated in any training related to volunteering?

Yes 1
No 2

I7. Please remember, have you ever registered with a volunteer service website or other organizations?

Yes 1
No 2
unclear........3

I8. What do you think are the shortcomings of the current voluntary service (multiple choices are allowed)?[Show the card on page 66]

Voluntary service projects are mainly allocated by League organizations, lacking autonomy 1

Inadequate plans and poor communication between organizers and volunteers 2

Volunteers lack enthusiasm..3

Lack of solid financial support 4

Formalism, not focusing on practical results 5

Volunteers cannot gain personal growth in the process of volunteer service.......6

There are no shortcomings..7

unclear 8

I9. For you and your family, which of the following volunteer services may be needed from others or organizations?(Multiple choices are allowed) [Show the card on page 67]

Care for children 01

Youth counselling 02

Elderly care 03

Women's rights protection...04

Help the disabled...05

Teaching assistant..06

Poverty alleviation...07

medical care 08

legal aid 09

environmental protection...10

Rescue and disaster relief...11

international aid 12

Others (please specify supporting the army and giving preferential treatment to families, educational aid, psychological consultation, large-scale social activities, etc.) 13

None of the above...14

[Note to investigators: please send thank-you letters and

This is the end of our visit. Thank you for your support to our
